# Environmental enrichment causes a global potentiation of neuronal responses across stimulus complexity and lamina of sensory cortex

**DOI:** 10.3389/fncel.2013.00124

**Published:** 2013-08-08

**Authors:** Dasuni S. Alwis, Ramesh Rajan

**Affiliations:** Department of Physiology, Monash UniversityClayton, VIC, Australia

**Keywords:** EE, barrel cortex, electrophysiology, hyperexcitability

## Abstract

Enriched social and physical housing produces many molecular, anatomical, electrophysiological and behavior benefits even in adult animals. Much less is known of its effects on cortical electrophysiology, especially in how sensory cortex encodes the altered environment, and extant studies have generally been restricted to neurons in input laminae in sensory cortex. To extend the understanding of how an enriched environment alters the way in which cortex views the world, we investigated enrichment-induced changes in neuronal encoding of sensory stimuli across all laminae of the rat barrel cortex receiving input from the face whisker tactile system. Animals were housed in Enriched (*n* = 13) or Isolated housing (*n* = 13) conditions for 8 weeks before extracellular recordings were obtained from barrel cortex in response to simple whisker deflections and whisker motions modeling movements seen in awake animals undertaking a variety of different tasks. Enrichment resulted in increases in neuronal responses to all stimuli, ranging from those modeling exploratory behavior through to discrimination behaviors. These increases were seen throughout the cortex from supragranular layers through to input Layer 4 and for some stimuli, in infragranular Layer 5. The observed enrichment-induced effect is consistent with the postulate that enrichment causes shift in cortical excitatory/inhibitory balance, and we demonstrate this is greatest in supragranular layers. However, we also report that the effects are non-selective for stimulus parameters across a range of stimuli except for one modeling the likely use of whiskers by the rats in the enriched housing.

## Introduction

Environmental enrichment (EE) for laboratory animals, in housing that allows more social interaction and cognitive and motor challenges compared with standard housing (Hebb, 1947; Van Praag et al., 2000; Nithianantharajah and Hannan, 2006), provides a remarkable array of cognitive and behavioral benefits in normal development and in adulthood (Rosenzweig and Bennett, 1996; Buonomano and Merzenich, 1998; Nilsson et al., 1999; Van Praag et al., 2000; Zimmermann et al., 2001; Lewis, 2004; Bruel-Jungerman et al., 2005; Li and Tang, 2005; Meshi et al., 2006; Nithianantharajah and Hannan, 2006; Sale et al., 2009; Veyrac et al., 2009).

Corresponding to these cognitive and behavioral changes, EE induces anatomical and molecular changes in the brain. The former include neuro-, glio-, synapto- and angio-genesis, decreased cell death, and increases in receptor numbers, transmitter synthesis, dendritic length, and branching, and thickness of the cerebral cortex (Holloway, 1966; Diamond et al., 1972; Globus et al., 1973; Greenough and Volkmar, 1973; Uylings et al., 1978; Sirevaag and Greenough, 1987; Rosenzweig and Bennett, 1996; Buonomano and Merzenich, 1998; Van Praag et al., 2000; Li and Tang, 2005; Nithianantharajah and Hannan, 2006; Sale et al., 2009), and increased levels of neurotransmitters, such as acetylcholine and noradrenaline, which promote neurogenesis and plasticity (Por et al., 1982; Rosenzweig and Bennett, 1996; Soares et al., 1999). Molecular changes include increases in levels of neurotrophic and growth factors, including brain-derived neurotrophic factor, nerve growth factor, and vascular endothelial growth factor which contribute to neuronal proliferation, development, signaling, survival, and ultimately, plasticity (Falkenberg et al., 1992; Pham et al., 1999; Ickes et al., 2000; During and Cao, 2006). Enhanced synaptogenesis after EE is coupled to an increase in synaptic proteins such as postsynaptic density-95 protein and synaptophysin (Frick and Fernandez, 2003; Nithianantharajah et al., 2004). EE-induced changes can occur rapidly—e.g., even structural changes (viz., number of excitatory and inhibitory synapses in Layer 4 of barrel cortex) can occur with just 24 h of EE (Landers et al., 2011).

Much less is known about EE effects on neuronal functionality, especially to behaviorally relevant inputs or outputs. Exposure to EE results in increased field potentials and greater excitatory post-synaptic potential slopes in *in vitro* hippocampal slice recordings (Sharp et al., 1985; Green and Greenough, 1986) and auditory cortex slices show increases in excitatory post-synaptic currents in layers 2/3, likely from enhanced glutamatergic transmission (Nichols et al., 2007). In extracellular recordings from auditory, visual and somatosensory cortices, EE effects include increased sensitivity and responsiveness in granular or supragranular layers (Engineer et al., 2004; Polley et al., 2004; Mainardi et al., 2010; Jakkamsetti et al., 2012; Tognini et al., 2012). Thus, in auditory cortex, there are significant increases in spontaneous and stimulus-evoked responses to sounds, longer response latency, and narrower receptive field (RF) bandwidths; additionally, improved temporal information processing is suggested by increased responsiveness to higher temporal modulation rates and increased paired-pulse depression (Engineer et al., 2004; Percaccio et al., 2005, 2007; Jakkamsetti et al., 2012). Somatosensory cortex maps are made more precise after EE through decreases in neuronal RF sizes (Polley et al., 2004; Frostig, 2006) and improved response selectivity (Coq and Xerri, 1998; Polley et al., 2004). In visual cortex, EE causes a decrease in cortical inhibition (Scali et al., 2012) promoting ocular dominance plasticity in aged rats, and accelerating development of visual cortex in animals exposed to EE from birth (Cancedda et al., 2004). The general view is that the core change induced by EE to produce these varied outcomes in cortical neurophysiology and functional encoding is a shift in cortical excitation/inhibition (E/I) ratios (Beaulieu and Colonnier, 1987; Coq and Xerri, 1998; Polley et al., 2004; Sale et al., 2009; Baroncelli et al., 2011 see Discussion). The great majority of studies of such cortical neuronal properties have been restricted to thalamo-recipient layers and little is known of changes in other layers, especially the supra-granular layers 2 and 3 (L2 and 3) that are considered to be a “privileged substrate” for consolidating EE-induced cortical plasticity (Nichols et al., 2007) with their great capacity for amplification of changes occurring in the thalamo-recipient layers (Komai et al., 2006; Brecht, 2007; Feldmeyer et al., 2013; Petersen and Crochet, 2013). The effects observed in thalamo-recipient layers may not simply predict effects in upper layers as indicated by the observation in the mature barrel cortex that deprivation-induced plasticity decreases Layer 4 feed-forward excitation to L2/3 inhibitory neurons but improves inhibition to L2/3 pyramidal cells (House et al., 2011), resulting in E/I balance being maintained in L2/3. Further the scattered body of data on the neurophysiological consequences of EE on cortex are generally limited to descriptions of RF sizes and response strength, generally to simple stimuli. We have now attempted to redress these deficits in knowledge by investigating EE related changes in circuit dynamics of neurons from supra-granular layer 2 through to infra-granular layer 5 of rat barrel cortex to simple stimuli and to complex stimuli that model the ways in which awake trained rats use their whiskers in many natural behaviors.

## Materials and methods

### Animals

Male Sprague–Dawley rats (aged ~7 weeks, weight 250 g) were obtained from Monash Animal Services (MAS) and littermates randomly assigned to either Isolated (Isol.; *n* = 13) or Enriched (EE; *n* = 13) housing conditions, always in a 12 h light/dark cycle with *ad libitum* food and water, for 8–10 weeks; from week 8 onwards, animals were removed for terminal electrophysiological experiments conducted over a 2 week period to ensure that there was no confound from ageing. All experiments were conducted in accordance with guidelines from the National Health and Medical Research Council and received approval from the Monash University Standing Committee on Ethics in Animal Experimentation.

### Housing conditions

Rats in EE housing were housed in groups of 3 in a large (69 × 60 × 270 cm) run which featured a front-facing Plexiglas wall and 3 steel walls with a steel base and a wire mesh cage lid. The cage floor was covered with wood shavings and contained numerous plastic and metal toys and objects of various colors, sizes, and textures. The objects used for enrichment were kept constant but their locations were re-arranged every 2.5 days. The Isolated housing rats were housed individually in standard sized cages (31× 24 × 45 cm) with wood shavings and shredded paper, but no toys.

### Extracellular recordings from barrel cortex

Cortical responses from animals in the two housing conditions were recorded 8–10 weeks after commencement of housing in the test condition. Extracellular recordings were taken from the posteromedial barrel subfield region of somatosensory cortex (PMBSF; the so-called barrel cortex), as detailed elsewhere (Rajan et al., 2006, 2007; Alwis et al., 2012). Briefly, animals were anesthetized with 5% halothane (Sigma Aldrich, USA) mixed in O_2_ (O_2_ flow rate = 1 ml/min) and tracheotomised for mechanical ventilation (2.5–3.5 mL tidal volume, 72–80 breaths/min; both dependent on animal size) to maintain anesthesia thereafter with 0.5–2% halothane mixed in O_2_ (O_2_ flow rate = 0.3 ml/min). A thermostatically-controlled heat pad with feedback from a rectal probe (Fine Science Tools Inc., U.S.A) was used to maintain body temperature at 37–38°C.

Once anesthesia was established, as determined from absent palpebral reflexes or responses to strong forepaw pinching, the skull was exposed and anchored to a head bar with a screw and dental cement. Then a section of skull ~5 mm in diameter, located above barrel cortex (~2 mm caudal of bregma and 6 mm lateral of the midline), was removed and the exposed cortex (dura intact) covered with silicone oil. Recordings were obtained using a parylene-coated tungsten microelectrode (2–4 MΩ resistance; FHC, ME, U.S.A) which was moved using a fast-stepping micro-drive (Kopf Instruments, California, U.S.A). Initially, the electrode was advanced to a depth between 600 and 800 μm from the surface to allow determination of the Principal Whisker (PW) by manual deflection of the whiskers using a hand held probe. Here the PW was classified as the whisker which produced the greatest neuronal firing response to manual whisker deflection (with electrode output monitored aurally through speakers as well as on an oscilloscope screen) and, in barrel cortex, was always unequivocally identifiable at this depth. Where drive was weak, or a result of multi-whisker activity, the electrode was retracted and a new penetration was made until a single PW could be identified; the electrode output was monitored (see below) after amplification and filtering, on an oscilloscope and through speakers. If strong PW drive was obtained (see Alwis et al., 2012 for details) the electrode was then retracted to the cortical surface under visual control and zeroed here. Then it was advanced systematically to record from neurons at a number of different depths, using stimuli delivered under computer control to the PW. The first recording was made at a depth of about 150 μm from the surface; thereafter recordings were made at regular intervals to ensure data was collected from all animals from the following layers: Layer 2 (150–300 μm); Upper Layer 3 (350–500 μm); Deep Layer 3 (550–700 μm); Layer 4 (750–1000 μm); and Layer 5 (1100–1400 μm). To ensure that data representation from deeper layers was not compromised by the later recordings perforce of this strategy, in some cases we advanced the electrode first to Layer 4 and collected data from that layer and then from Layer 5 before retracting back to the surface to advance systematically as above to obtain data from the other layers. We have previously demonstrated that recorded responses in halothane anesthetized animals, using the same experimental techniques outlined in the present study and in our previous work (see Rajan et al., 2006; Alwis et al., 2012; Johnstone et al., 2013), were comparable to those seen in awake animals, where the pattern of temporal and spatial responses to whisker deflection were similar in both the anesthetized and awake states (Rajan et al., 2006; Maravall et al., 2007).

Neural signals from the microelectrode were treated as described previously (see Rajan et al., 2006, 2007; Alwis et al., 2012 for details), being amplified, band-pass filtered (0.3–10 kHz) and displayed on an oscilloscope, and through speakers for aural monitoring of neural activity. A Schmitt trigger box was used to set a voltage trigger level, while monitoring on the oscilloscope at a level 2× noise level, for neuronal cluster activity recordings. Trigger crossings generated digital pulses that were fed to a PC; this computer used Spike 2 software (CED, UK) for stimulus generation and/or delivery (Alwis et al., 2012) and stimulus trigger events were stored along with the Schmitt trigger pulses. The Spike2 software was also used to generate on-line displays of rasters of spike occurrences and peristimulus time histograms (PSTHs). A minimally-filtered copy of the signals recorded by the electrode was also stored for any offline analysis if needed later (Rajan et al., 2006).

### Controlled whisker deflections for quantitative barrel cortex data collection

For quantitative data recording, the PW was threaded through a hole at the end of a motor-controlled lever arm system positioned 5 mm from the mystacial pad to deflect the PW in computer-controlled patterns. At each recording site, voltage triggers were set at a level 2× noise level to obtain responses from neuronal clusters (of 4–6 neurons, as determined by online spike sorting using Spike 2 software). Responses were first characterized for laminar location using a suite of 3 trapezoidal stimuli, where only the onset ramp velocity was varied (60, 150, 400 mm/s; Alwis et al., 2012). This suite of trapezoids was presented for 150–300 repetitions in a pseudo-random manner.

Then a series of four complex “naturalistic” whisker deflections, obtained from studies in which whisker motion was recorded in awake behaving rats, were played out from text files which stored stimulus characteristics. These stimuli have been described in detail in our previous studies (Alwis et al., 2012; Johnstone et al., 2013); the four waveforms were those seen in whisker motion across a smooth and rough surface (Ritt et al., 2008); when rats made contact with a rod placed in the path of the whiskers (Hartmann et al., 2003); and in head-fixed rats that were engaging in “free” whisking (Gao et al., 2001). Details of the acquisition and conversion of these waveforms to whisker deflection patterns have been previously described by our group (Alwis et al., 2012; Johnstone et al., 2013). Each suite consisted of 10 stimulus amplitudes, from the lowest amplitude of 0.2 mm, and then continuing from 0.4 to 3.6 mm in 0.4 mm steps. Each suite of 10 stimulus amplitude was presented 50 times, in pseudo-random order across successive presentations.

### Data analysis

Cluster responses were segregated into lamina by depth (from the cortical surface) as noted above: Layer 2 (150–300 μm); Upper Layer 3 (350–500 μm); Deep Layer 3 (550–700 μm); Layer 4 (750–1000 μm); and Layer 5 (1100–1400 μm). Data were represented as firing rate (in spikes/s) in 1 ms bins over the period from 200 ms prior to stimulus onset until 100 ms post stimulus offset. The data collected in the 200 ms pre-stimulus bin was used to calculate spontaneous firing rates, which were subtracted from responses during the rest of the data collection period to correct for the spontaneous firing rate. The spontaneous activity-corrected firing rates were used for all subsequent analyses.

Offline analysis was conducted to generate population peri-stimulus time histograms (PSTHs) showing the pattern of population responses within a lamina, in animals housed in either Enrichment or Isolation. PSTHs were obtained by averaging cluster responses within a lamina across each presentation of a stimulus. A 5-point weighted moving average was then applied to the data to smooth out noise and a grand PSTH was produced by averaging the data across all multi-units.

For quantitative analysis for each stimulus, we used only data from multi-neuronal clusters considered to be responsive to that stimulus. Clusters were classified as responsive if their response rates were significantly greater (more than 1.4 *SD* >) than spontaneous firing rates at more than two consecutive stimulus velocities (for the trapezoidal stimuli) or at more than two consecutive stimulus amplitudes (for the four naturalistic stimuli). We then extracted the following metrics for each stimulus: peak firing rate, area under the curve, latency to peak and half-peak width. These calculations were done using specific counting windows for each stimulus: for the trapezoidal and object contact stimuli, a counting window from 5 to 50 ms after stimulus onset was used, for the surface texture discrimination stimuli, a 5–30 ms counting window, while the free whisking stimulus was analysed using a 5–200 ms counting window. Almost identical effects were seen for the firing rate measures (peak firing rate and area under the curve), and almost identical effects for the timing measures (latency to peak and half-peak width) and hence, we present data only from the peak response (PFR) and the latency to the PFR (*L*_PFR_) for each stimulus in its designated analysis window.

Statistical analysis was carried out using two-way repeated measures ANOVAs to determine laminar-specific differences, with peak firing rates or latencies being the dependent variable and the independent between-animal factors being housing condition and stimulus amplitude/velocity. When the ANOVA revealed a significant main factor effect of Group (housing condition) or Stimulus parameter (Amplitude/Velocity) or a significant interaction term between these two factors, *post-hoc* Bonferroni tests were used to where the differences lay.

## Results

### Database and measures of neuronal responses

Electrophysiological recordings were obtained from 13 rats housed in Isolation and 13 rats in EE housing, from multi-unit clusters in layers 2 (L2), Upper layer 3 (U3), Deep layer 3 (D3), Layer 4 (L4), and Layer 5 (L5), in response to simple and complex, “naturalistic” whisker motion patterns. Only data from responsive multi-neuronal clusters (see Materials and Methods for definition) were extracted for analysis. We present below, for each stimulus, laminar-specific data on the response patterns of clusters in the two housing conditions, and then data for two major metrics of the responses.

To visualize the laminar-specific response pattern of neuronal clusters to any specific stimulus, Grand Peri-Stimulus Time Histograms (GPSTHs), which are defined as histograms of firing rate against time from stimulus onset, were generated. For this, responses of each cluster during and after the stimulus period were corrected for the spontaneous activity measured in that cluster in the 200 ms period prior to stimulus onset. Then, for rats in each housing condition, responses from all responsive clusters in a specific lamina were averaged to generate the GPSTH of firing rate against stimulus period for that lamina. Finally, to quantify the effects, analysis windows were defined for each stimulus in relation to aspects of that stimulus (e.g., the onset period, or the post-stimulus offset period; defined in Materials and Methods for each stimulus), and for these windows we present two representative quantitative metrics from all responsive clusters: (1) peak excitatory firing rate (PFR) and (2) latency to the peak (*L*_PFR_). The PFR was the across-clusters average peak firing rate during the stimulus period as seen in the population GPSTH and expressed as spikes/s, and the *L*_PFR_ was the time from stimulus onset to this peak in the GPSTH. Note that other firing rate and timing values were also calculated (see Materials and Methods) but the effects were the same across all firing rate measures or across all timing measures and hence only PFR and *L*_PFR_ are presented here.

### Changes in neuronal responses to complex whisker stimuli after exposure to EE

#### Population responses to a simple whisker motion stimulus

***Trapezoidal whisker displacements***. The first stimulus applied to the PW of each cluster was a suite of 3 trapezoid stimuli, with one of three onset ramp (whisker protraction) velocities (60, 150, and 400 mm/s) which we have shown (Rajan et al., 2007) to elicit population responses that cover the range from low neural response rates (velocity of 60 mm/s) to saturation of neural responses (velocity of 400 mm/s). Data were obtained to these stimuli from 86 responsive clusters in Isolation-housed animals and 84 responsive clusters in EE animals; there was no difference between the two housing conditions in the number of responsive clusters per layer (χ^2^ = 3.6, *df* = 4, *p* > 0.05).

**Figure 1:**
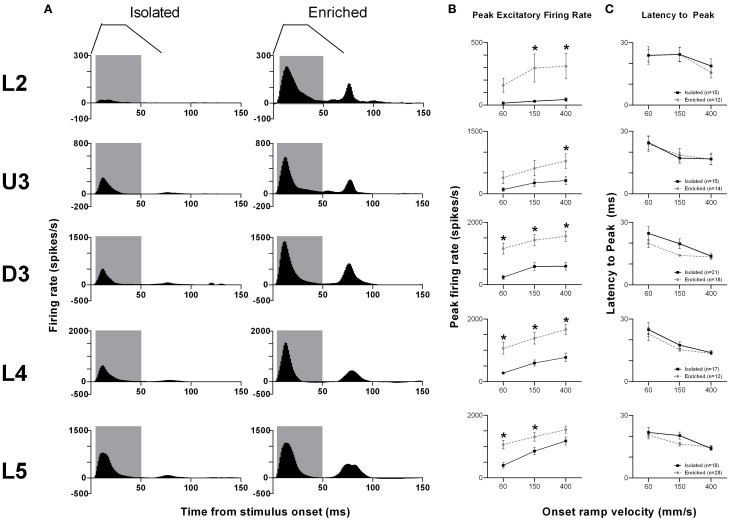
**EE effects on pattern, strength and timing of responses evoked by simple trapezoidal stimuli**. For this simple stimulus, response strength significantly increased in all laminae after EE, with no changes in response timing. **(A)** Population Grand PSTHs in response to the trapezoid with the fastest onset ramp velocity (400 mm/s) in a specific lamina (indicated to left of panel) in EE and Isolated animals. Laminar designations: L2, Layer 2; U3, Upper Layer 3; D3, Deep Layer 3; L4, Layer 4; L5, Layer 5. Each Grand PSTH was generated by averaging responses across all responsive clusters in that lamina. The analysis window used to extract response metrics is represented by the gray shaded box (5–50 ms). Stimulus waveform is presented above both panels. **(B)** Peak firing rate (PFR) and **(C)** Latency to peak (*L*_PFR_) extracted from the onset response to simple trapezoidal stimuli from clusters in EE animals (gray circles) and in Isolated animals (black squares). Data represents averages from all responsive clusters (±SEM) at all tested ramp velocities, separated by cortical lamina. Each row of data comes from the same lamina as designated by the labels on the left; cluster numbers for each layer are listed in the key in the last column. ^*^p < 0.05.

The pattern of population responses to the trapezoid stimuli are exemplified in Figure 1A by the GPSTHs from the highest onset ramp velocity of 400 mm/s for all cortical laminae. In Isolated housing animals, responses to the trapezoid consisted of a well-defined response during the onset ramp, evident in all layers, and a second peak, poorly defined in all layers, corresponding to stimulus offset. The GPSTHs from EE animals showed the same two peaks, but with the offset response now being well-defined, and with a marked increase in firing rates in all cortical layers. Increases in peak firing rate were observed for both onset and offset responses, while tonic excitation in the period between the onset and offset responses was also increased in the supragranular L2–D3 in EE animals.

An analysis window of 5–50 ms from stimulus onset, covering the entire onset peak response at all three onset ramp velocities, was used to obtain quantitative metrics on response strength (Peak firing rate, PFR, in the onset response; Figure 1B) and timing (Latency to Peak firing, *L*_PFR_; Figure 1C). Each dataset was analysed using Two-Way repeated measures ANOVAs, with housing condition and lamina being between-subjects variables and onset ramp velocity being the within-subjects variable. In all cortical layers, the PFRs in EE animals were significantly higher than PFRs in Isolation-housed animals (*p* < 0.05; see Supplementary Table S1 for statistical details). In L2, a significant increase in firing rate was seen at the highest two velocities; in U3 only at the highest velocity; at all velocities in Deep Layer 3 and 4; and at the two lowest velocities in L5 (see Supplementary Table S1 for details: Two-Way ANOVA, Bonferroni *post-hoc*, *p* < 0.05). However, this EE-related marked increase in firing strength in all layers occurred with no changes in the latency to the peak onset response, *L*_PFR_ (Figure 1C) at any onset ramp velocity in any cortical layer (*p* > 0.05; see Supplementary Table S1).

#### Responses to complex whisker motion stimuli

***Object contact stimulus***. Hartmann et al. (2003; Figure 8A from Hartmann paper) imaged whisker motion in an unrestrained rat that was using its whiskers to brush past a metal post to obtain a liquid reward. We extracted a significant segment of this “object contact” stimulus waveform (see Materials and Methods; Alwis et al., 2012) and applied this segment to the PW in recordings from 79 responsive clusters in Isolation-housed animals and 82 responsive clusters in EE animals. There was no difference between the two housing groups in numbers of responsive clusters in each layer (χ^2^ = 3.5, *df* = 4, *p* > 0.05).

**Figure 2:**
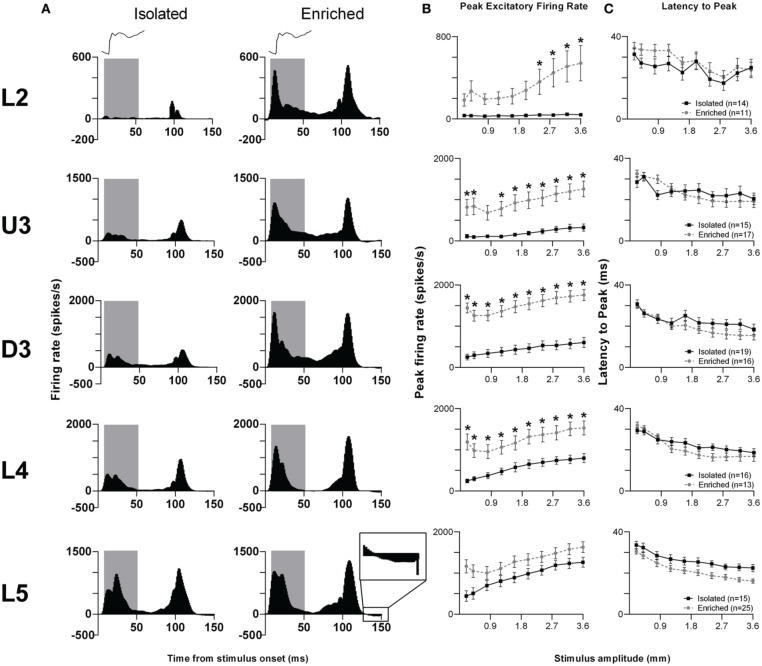
**EE effects on pattern, strength and timing of responses evoked by the object contact stimulus**. For this complex stimulus, response strength significantly increased in all layers from Layer 2 to Layer 4 after EE, with no changes in response timing. **(A)** Population Grand PSTHs in response to the trapezoid with the fastest onset ramp velocity (400 mm/s) in a specific lamina (indicated to left of panel) in EE and Isolated animals. Laminar designations: L2, Layer 2; U3, Upper Layer 3; D3, Deep Layer 3; L4, Layer 4; L5, Layer 5. Each Grand PSTH was generated by averaging responses across all responsive clusters in that lamina. The analysis window used to extract response metrics is represented by the gray shaded box (5–50 ms). Stimulus waveform is presented above both panels. Inset shows a magnified view of post-stimulus inhibition **(B)** Peak firing rate (PFR) and **(C)** Latency to peak (*L*_PFR_) extracted from the onset response to simple trapezoidal stimuli from clusters in EE animals (gray circles) and in Isolated animals (black squares). Data represents averages from all responsive clusters (±SEM) at all tested stimulus amplitudes, separated by cortical lamina. Each row of data comes from the same lamina as designated by the labels on the left; cluster numbers for each layer are listed in the key in the last column. ^*^p < 0.05.

We illustrate the pattern of neuronal responses to this complex stimulus using Grand PSTHs (GPSTHs) obtained at the highest stimulus amplitude (3.6 mm) from all responsive clusters in a layer for rats in a particular housing (Figure 2A). In Isolation-housed animals, the GPSTH generally consisted of two response peaks corresponding to stimulus onset and offset, with low levels of tonic excitation in between. Specifically, GPSTHs from L2 and U3 showed poorly defined onset responses but larger, more defined peaks at stimulus offset (Figure 2A), whereas in D3, L4, and L5, clearly-defined onset and offset responses were present, with the onset response consisting of two peaks for all eight stimulus amplitudes >0.4 mm whisker deflection. Following the stimulus offset response in L4 and L5, responses decreased to below spontaneous activity rates (recorded in the 200 ms pre-stimulus period) and were classified as inhibitory responses. This same general pattern of activity was seen in EE animals but with a marked increase in onset and offset excitation in all layers except L5. Post-offset inhibition, as evidenced by a decrease in firing rate below spontaneous firing rate was now clearly evident both during and after the stimulus in all layers except L2. In general, compared to neuronal responses in Isolation-housed animals, EE animals showed increased excitation in L2–4 for onset and offset responses, and minor changes in L5.

Quantitative metrics of population responses to the object contact stimulus were extracted using an analysis window of 5–50 ms from stimulus onset, encompassing the entire onset response. For the peak excitatory firing rate (PFR) in this onset window (Figure 2B), Two-Way repeated measures ANOVAs (between-subjects variables: housing condition and lamina; within-subjects variable: stimulus amplitude) revealed significant differences (*p* < 0.05; see Supplementary Table S2 for statistical details) between Isolated housing and EE animals in L2–4 but no differences in L5. The PFR was higher in EE animals than in Isolated housing animals in all layers, with significant increases at the four highest stimulus amplitudes in L2 (2.4–3.6 mm), at all but one intermediate amplitude (0.8 mm) in U3, and at all amplitudes in D3 and L4 (Two-Way ANOVA, Bonferroni *post-hoc*, *p* < 0.05). For the onset response timing, measured as the latency to the onset peak (*L*_PFR_), Two-Way ANOVAs found no significant differences between Isolation-housed and EE animals in L2–4 (*p* < 0.05; Figure 2C; Supplementary Table S2): in both housing conditions *L*_PFR_ decreased systematically with increasing stimulus amplitude. There was a significant housing condition effect but no housing × amplitude interaction in L5, reflecting a shorter *L*_PFR_ in EE animals when compared with Isolation-housed animals, at all stimulus amplitudes.

Thus, as in the case of the simpler trapezoid stimuli, for this “object contact” stimulus waveform, response strength in L2–4 was greater at all or most stimulus amplitudes in EE animals than in Isolated housing controls. Generally, there were no differences in response timing between Isolation-housed and EE animals in all layers except L5, where *L*_PFR_ was found shorter in EE animals.

***Surface texture discrimination stimuli***.

*Smooth surface discrimination whisker motion*. As in our previous studies (Alwis et al., 2012; Johnstone et al., 2013) our suite of complex whisker stimuli included two motion patterns (recorded by Ritt et al., 2008) which mimic whisker motion in awake, unrestrained rats trained to discriminate between smooth and rough surface textures. First, we consider the responses to the whisker stimulus which mimics a significant portion of whisker motion across a smooth surface (Ritt et al., 2008). This stimulus was used while recording from 78 responsive clusters in Isolation-housed animals and 85 responsive clusters in EE animals (no difference between groups in number of responsive clusters in every layer: χ^2^ = 3.5, *df* = 4, *p* > 0.05).

**Figure 3:**
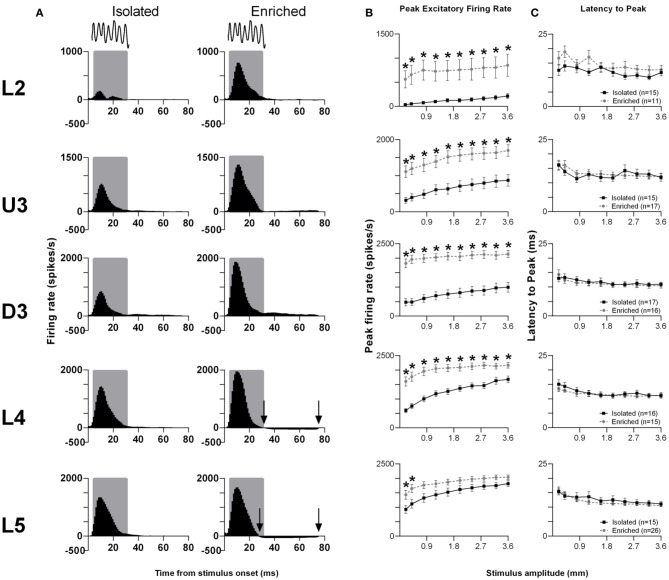
**EE effects on pattern, strength and timing of responses evoked by the smooth surface discrimination stimulus**. Response strength significantly increased in all cortical laminae after EE, with no changes in response timing. **(A)** Population Grand PSTHs in response to the trapezoid with the fastest onset ramp velocity (400 mm/s) in a specific lamina (indicated to left of panel) in EE and Isolated animals. Laminar designations: L2, Layer 2; U3, Upper Layer 3; D3, Deep Layer 3; L4, Layer 4; L5, Layer 5. Each Grand PSTH was generated by averaging responses across all responsive clusters in that lamina. The analysis window used to extract response metrics is represented by the gray shaded box (5–30 ms). Stimulus waveform is presented above both panels. Black arrows highlight periods of post-stimulus inhibition. **(B)** Peak firing rate (PFR) and **(C)** Latency to peak (*L*_PFR_) extracted from the onset response to simple trapezoidal stimuli from clusters in EE animals (gray circles) and in Isolated animals (black squares). Data represents averages from all responsive clusters (±SEM) at all tested stimulus amplitudes, separated by cortical lamina. Each row of data comes from the same lamina as designated by the labels on the left; cluster numbers for each layer are listed in the key in the last column. ^*^p < 0.05.

The pattern of responses to this complex stimulus from all responsive clusters in a layer for rats in the two housing conditions is seen in Grand PSTHs (GPSTHs) to the highest stimulus amplitude (3.6 mm; Figure 3A). Generally, response patterns were similar in both groups, with a peak of firing corresponding to stimulus onset. In Isolated housing animals, this peak was followed by low levels of tonic excitatory activity in all cortical layers, while in EE animals, post-offset response inhibition (response rate < spontaneous response rate) was seen instead of tonic excitation in L4 and L5. Response strength was markedly increased in EE animals in supragranular layers (L2–D3); while in granular and infragranular layers (L4 and L5), differences in response strength between groups were more evident at lower amplitudes, and became less obvious at higher stimulus amplitudes. The PFR and *L*_PFR_ were obtained using an analysis window of 5–30 ms which covered the entire stimulus duration. Analysis of PFR (Two-Way repeated measures ANOVAs; between-subjects variables: housing condition and lamina; within-subjects variable: stimulus amplitude) found significant group differences in all layers (Figure 3B, *p* < 0.05; see Supplementary Table S3 for statistical detail), with PFRs in EE animals being higher than those of Isolation-housed animals. *Post-hoc* analysis showed that the significant increases in PFRs in EE animal occurred across all amplitudes in L2–4 (*p* < 0.05); in L5, PFRs in EE animals were only significantly higher than those in Isolation-housed animals at the two lowest amplitudes (0.2 and 0.4 mm). As with the other stimuli detailed above, there were no significant changes in onset peak timing (*L*_PFR_; Figure 3C) between the two housing conditions in all layers (*p* > 0.05; see Supplementary Table S3 for further details); in both groups there was a significant systematic decrease in *L*_PFR_ with increasing stimulus amplitude.

**Figure 4:**
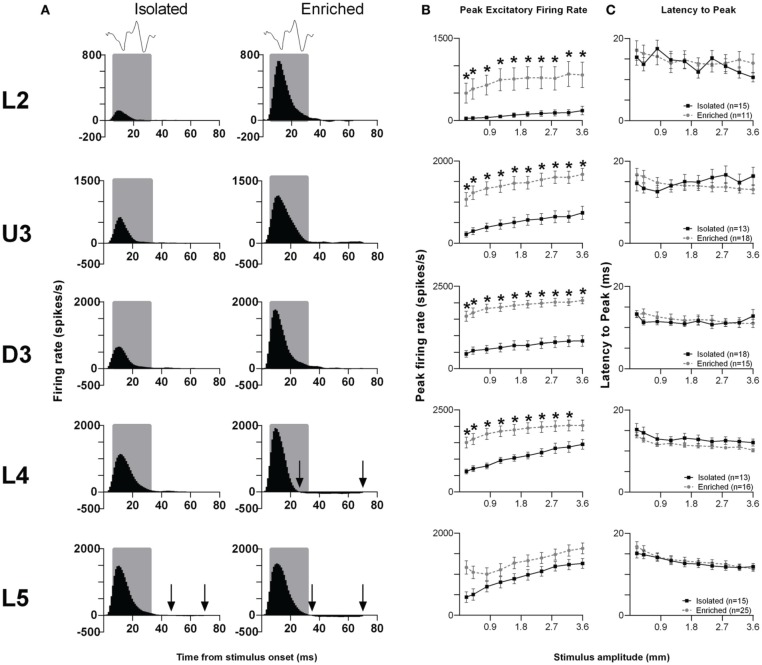
**EE effects on pattern, strength and timing of responses evoked by the rough surface discrimination stimulus**. Significant increases in response strength were found in L2–4 after EE (but not L5), with no changes in response timing. **(A)** Population Grand PSTHs in response to the trapezoid with the fastest onset ramp velocity (400 mm/s) in a specific lamina (indicated to left of panel) in EE and Isolated animals. Laminar designations: L2, Layer 2; U3, Upper Layer 3; D3, Deep Layer 3; L4, Layer 4; L5, Layer 5. Each Grand PSTH was generated by averaging responses across all responsive clusters in that lamina. The analysis window used to extract response metrics is represented by the gray shaded box (5–30 ms). Stimulus waveform is presented above both panels. Black arrows highlight periods of post-stimulus inhibition. **(B)** Peak firing rate (PFR) and **(C)** Latency to peak (*L*_PFR_) extracted from the onset response to simple trapezoidal stimuli from clusters in EE animals (gray circles) and in Isolated animals (black squares). Data represents averages from all responsive clusters (±SEM) at all tested stimulus amplitudes, separated by cortical lamina. Each row of data comes from the same lamina as designated by the labels on the left; cluster numbers for each layer are listed in the key in the last column. ^*^p < 0.05.

*Rough surface discrimination whisker motion*. The second of the two discrimination stimulus waveforms we used was a whisker motion pattern which mimicked the movement of whiskers across a rough surface (Ritt et al., 2008), characterized by a number of “stick-slip” events (Wolfe et al., 2008). For this stimulus, we recorded from 74 clusters in Isolation-housed animals and 85 clusters in EE animals (cluster numbers not significantly different between groups and layers: χ^2^ = 3.7, *df* = 4, *p* > 0.05). Response patterns were similar to those elicited to the smooth surface discrimination stimulus (Figure 4A), as were measures of firing rate (Figure 4B; see Supplementary Table S4 for further details) and response timing (Figure 4C; Supplementary Table S4 for further details).

In summary, for both discrimination stimuli, in L2–4 EE animals showed an increase in PFR compared with Isolation-housed animals, with no changes in the timing of responses.

***Exploratory “free whisking” stimulus***. The last complex stimulus waveform we consider is one that models the movement of rat whiskers when undergoing normal exploratory whisking (Gao et al., 2001), characterized by two cycles of sinusoid-like back and forth motion. Data for this stimulus waveform were obtained from 72 responsive multi-unit clusters in Isolation-housed animals and 84 multi-unit clusters in EE animals (cluster numbers not significantly different between groups and layers: χ^2^ = 3.6, *df* = 4, *p* > 0.05).

**Figure 5:**
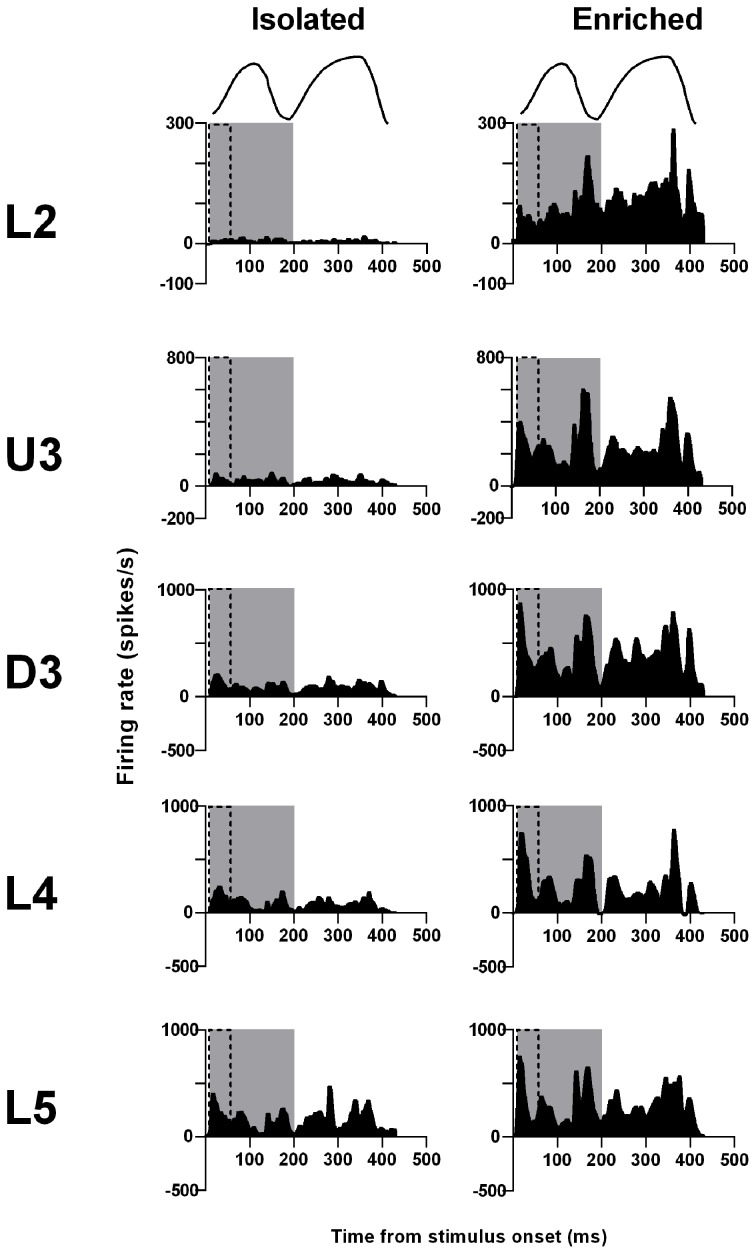
**EE effects on pattern of responses evoked by the free whisking stimulus**. Response strength increased and was more precisely defined across all laminae after EE. Population Grand PSTHs in response to the trapezoid with the fastest onset ramp velocity (400 mm/s) in a specific lamina (indicated to left of panel) in EE and Isolated animals. Laminar designations: L2, Layer 2; U3, Upper Layer 3; D3, Deep Layer 3; L4, Layer 4; L5, Layer 5. Each Grand PSTH was generated by averaging responses across all responsive clusters in that lamina. The two analysis windows used to extract response metrics are represented by the gray shaded box (5–200 ms) and the black dashed box (5–50 ms). Stimulus waveform is presented above both panels.

The laminar-related pattern of responses to this stimulus is shown in Grand PSTHs (GPSTHs) from the highest stimulus amplitude (3.6 mm) from all responsive clusters in a layer (Figure 5). In L2 and U3, responses in Isolation-housed animals consisted of low level excitation that was poorly defined throughout the stimulus. In deeper layers (D3–L5), responses in Isolation-housed animals were better defined as consisting of several peaks which appeared to coincide with changes in velocity in the stimulus waveform. In contrast, responses in the superficial layers (L2 and U3) in EE animals showed an overall increase in excitatory activity throughout the stimulus duration, resulting in responses being much better defined and consisting of a number of clear peaks appearing to align with velocity changes. Deeper layers (D3–L5) exhibited well-defined responses and similar response patterns to those in Isolated housing animals, albeit with much higher firing rates.

**Figure 6:**
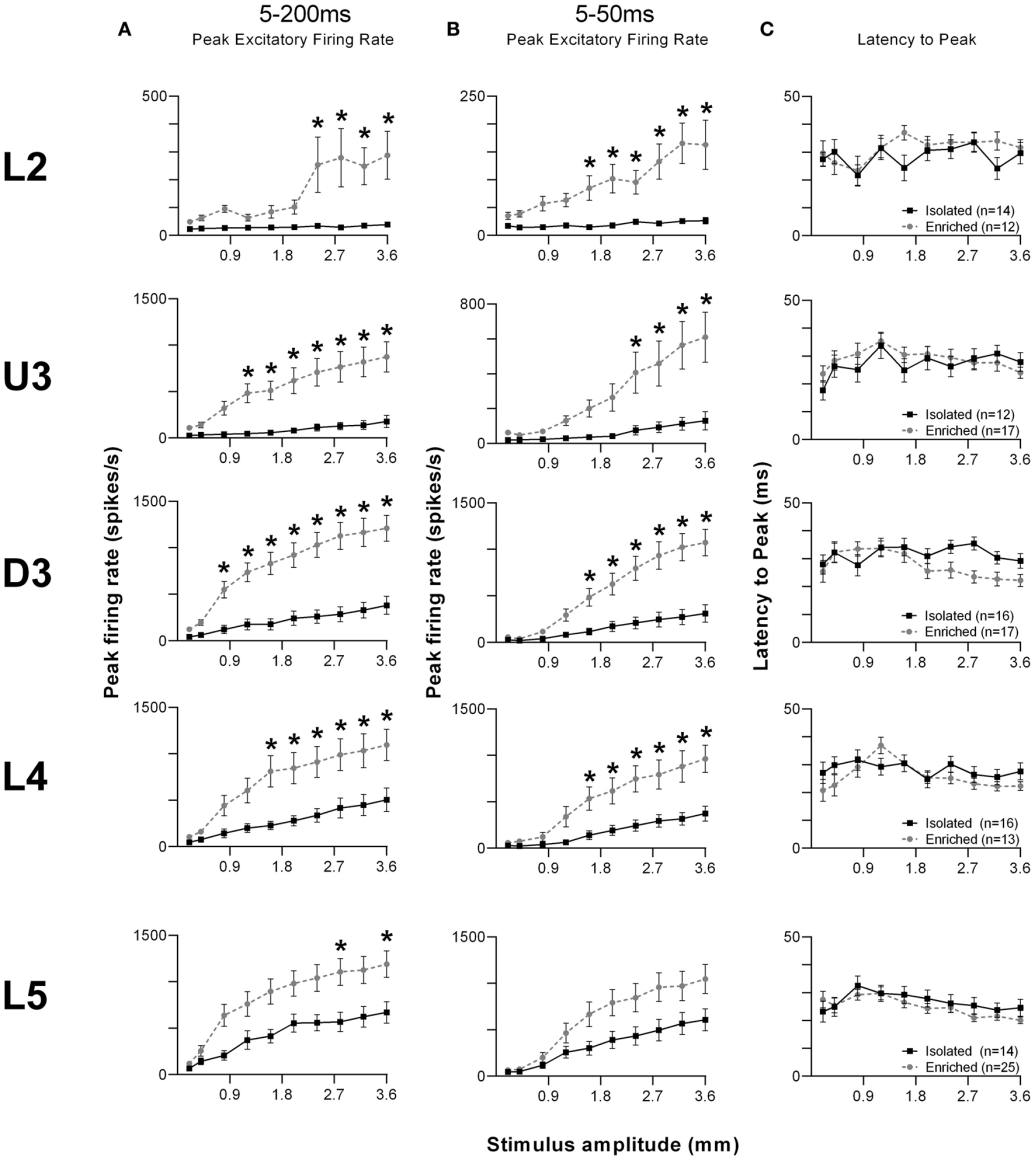
**EE effects on strength and timing of responses evoked by the free whisking stimulus. (A)** Peak firing rate (PFR) extracted from a 5–200 ms window, **(B)** PFR and **(C)** Latency to peak (LPFR) extracted from a 5–50 ms window in response to the free whisking stimulus from clusters in EE animals (grey circles) and in Isolated animals (black squares). PFR responses were significantly increased across L2–4 for both analysis windows, with a significant PFR increase in L5 only for the 5–200 ms window. No changes in response timing were found. Data represents averages from all responsive clusters (±SEM) at all tested stimulus amplitudes, separated by cortical lamina. Laminar designations: L2, Layer 2; U3, Upper Layer 3; D3, Deep Layer 3; L4, Layer 4; L5, Layer 5. Each row of data comes from the same lamina as designated by the labels on the left; cluster numbers for each layer are listed in the key in the last column. ^*^p < 0.05.

Given the complex nature of the response pattern, we first used an analysis window of 5–200 ms post-stimulus onset, to study changes in PFR throughout the stimulus (Figure 6A). Laminar-specific Two-Way repeated measures ANOVAs (details in Supplementary Table S5) found significant differences in PFR as a function of housing condition, and significant housing × amplitude interactions in L2–5, where again, PFRs in EE animals were higher than those in Isolated housing animals. *Post-hoc* analysis revealed that in L2, increased responses in EE animals were confined to higher stimulus amplitudes (2.4–3.6 mm), while in U3, D3, and L4, responses in EE animals were significantly higher at mostly higher stimulus amplitudes (Bonferroni *post-hoc*, *p* < 0.05). L5 responses were significantly higher in EE animals at only two of the highest stimulus amplitudes (2.8 and 3.6 mm; Bonferroni *post-hoc*, *p* < 0.05).

To allow comparison to metrics obtained to the other stimuli using shorter analysis windows, we then used a shorter analysis window (5–50 ms) to characterize changes in PFR and *L*_PFR_ during the onset ramp component of the stimulus (Figures 6B,C). Laminar-specific Two-Way repeated measures ANOVAs (see Supplementary Table S5 for further details) found significant differences in PFR as a function of housing condition and significant housing × amplitude interactions in L2–4, where responses in EE animals were higher than those in Isolation-housed animals but only at the higher amplitudes (Bonferroni *post-hoc*, *p* < 0.05; Figure 6B). In L5 there was no effect of housing condition but there was a significant Group × Amplitude interaction (Two-Way ANOVA, *p* < 0.05). Finally, analysis of *L*_PFR_ data from each lamina (Figure 6C) found a significant Group × Amplitude interaction only in D3, with no significant group effects in any layer (*p* > 0.05; statistical details in Supplementary Table S5).

In summary, in L2–4 the effects of housing on neuronal cluster responses for this stimulus was similar to the effects with the surface discrimination stimuli: EE animals showed an increase in PFR compared with Isolation-housed animals, with no changes in timing of responses.

#### Comparison of the effect sizes to the different stimuli in the different laminae

**Figure 7:**
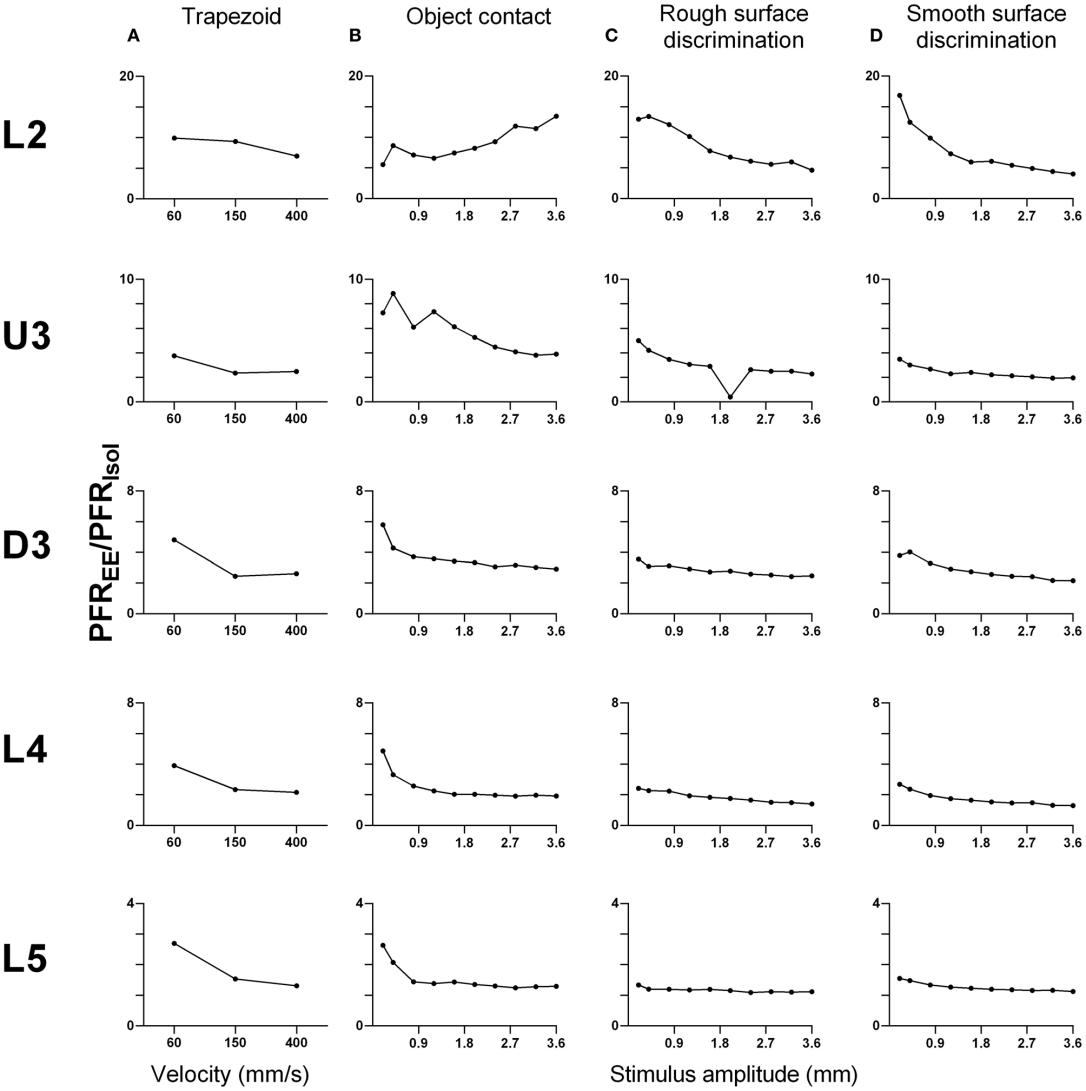
**Peak firing rate ratios in EE vs. Isol animals for trapezoid, object contact and surface discrimination stimuli**. PFR_EE_/PFR_Isol_ across all onset ramp velocities in **(A)** a simple trapezoidal stimulus; across all stimulus amplitudes in **(B)** the object contact stimulus, **(C)** the smooth surface discrimination stimulus and **(D)** the rough surface discrimination stimulus. PFR_EE_/PFR_Isol_ was greatest at lower velocities/amplitudes and decreased with increasing velocity/amplitude in all cortical layers as responses in EE animals came closer to those in Isolation-housed animals. Laminar designations: L2, Layer 2; U3, Upper Layer 3; D3, Deep Layer 3; L4, Layer 4; L5, Layer 5. Each row of data comes from the same lamina as designated by the labels on the left.

**Figure 8:**
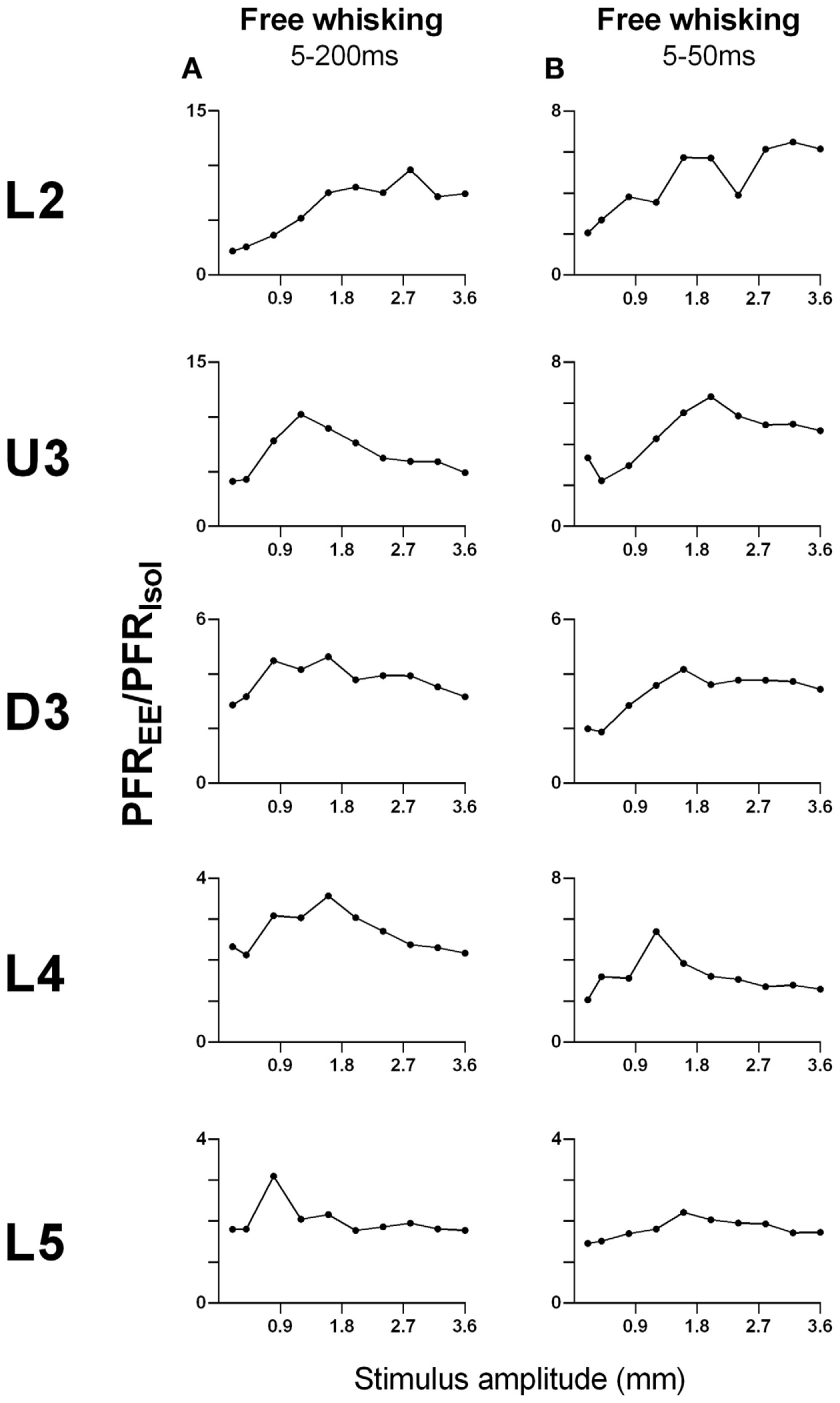
**Peak firing rate ratios in EE vs. Isol animals for free whisking stimulus**. PFR_EE_/PFR_Isol_ across all stimulus amplitudes in **(A)** the 5–200 ms analysis window, and **(B)** the 5–50 ms analysis window of the free whisking stimulus. For the 5–200 ms analysis window, there was a non-monotonic increase in PFR_EE_/PFR_Isol_, especially in layers U3–L5. For the 5–50 ms window, the PFR_EE_/PFR_Isol_ also increased non-monotonically and was followed by a plateau/decrease in magnitude of increased responsiveness in EE vs. Isolated animals. Laminar designations: L2, Layer 2; U3, Upper Layer 3; D3, Deep Layer 3; L4, Layer 4; L5, Layer 5. Each row of data comes from the same lamina as designated by the labels on the left.

As shown above, EE induced an increase in firing rate across all stimuli for most laminae. To make a comparison of the size of the EE-induced effects, for each stimulus type we calculated the ratio of PFR in EE animals for a specific stimulus condition (stimulus type × velocity/amplitude) vs. PFR for the same condition for clusters from Isolation-housed animals. This PFR_EE_/PFR_Isol_ ratio is shown in Figure 7 for four stimuli (trapezoid whisker deflections, the two surface discrimination stimuli, and the object contact stimulus) and in Figure 8 for the exploratory whisking stimulus.

A striking feature for the first four stimuli is that, aside from L2 responses to one stimulus (Figure 7B), a similar pattern of effects is seen in all laminae. In all cases, EE animals showed greater responses than Isolation-housed animals but the increase was inversely related to the appropriate stimulus parameter (trapezoids: onset ramp velocity; all other stimuli: stimulus amplitude)—i.e., the PFR_EE_/PFR_Isol_ was greater at lower velocities/amplitudes and decreased with increasing velocity/amplitude in all cortical layers. At the highest velocity/amplitude increased, responses in EE animals came closer to those in Isolation-housed animals. This pattern was consistent across the four very different stimuli, ranging from simple trapezoids to three of the complex, naturalistic stimuli.

However, a very different pattern of effects was seen for the PFR_EE_/PFR_Isol_ ratio to the exploratory whisking stimulus (Figure 8). Using a 5–200 ms analysis window to cover the whole stimulus, the PFR_EE_/PFR_Isol_ *increased* with stimulus amplitude but non-monotonically (Figure 8A), especially in layers U3–L5, where the greatest PFR_EE_/PFR_Isol_ is at intermediate amplitudes and decreased toward 1 at higher amplitudes. When the analysis window was restricted to only the “onset ramp” of the sinusoid-like stimulus (5–50 ms), a window similar to that used for the other stimuli (see above), the PFR_EE_/PFR_Isol_ still showed non-monotonicity, with maximum difference between EE and Isolated groups at intermediate stimulus amplitudes, followed by a plateau or even a decrease in magnitude of increased responsiveness in EE relative to Isolated animals (Figure 8B). Again, at high amplitudes responses in EE animals approached the peak firing rates of Isolated animals.

**Figure 9:**
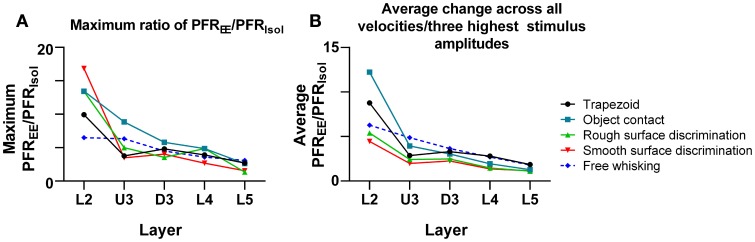
**Depth-dependent changes in maximum and average PFR_EE_/PFR_Isol_ ratios. (A)** Maximum PFR_EE_/PFR_Isol_ ratio in all stimuli plotted across all cortical layers. The maximum PFR_EE_/PFR_Isol_ ratio is in supragranular layers for all stimuli, and steadily declines as cortical depth increases. **(B)** Average PFR_EE_/PFR_Isol_ ratio in all stimuli from all onset ramp velocities/3 highest stimulus amplitudes, plotted across all cortical layers. The average PFR_EE_/PFR_Isol_ ratio is highest in supragranular layers for all stimuli, and decreases as cortical depth increases. Laminar designations: L2, Layer 2; U3, Upper Layer 3; D3, Deep Layer 3; L4, Layer 4; L5, Layer 5. Symbols corresponding to the five stimuli are presented in the keys to the right of the figure.

One other feature evident in the previous two figures is that the largest PFR_EE_/PFR_Isol_ ratio (i.e., the greatest increase in firing rate in EE animals relative to firing rate in Isolation-housed animals) appeared to occur in supra-granular layers. To demonstrate this, Figure 9A plots the maximum ratio of PFR_EE_/PFR_Isol_ for all the stimuli; to demonstrate that the effects were not distorted by a singular maximum value, Figure 9B plots the average PFR_EE_/PFR_Isol_ ratio calculated across the three highest stimulus amplitudes (for the four complex naturalistic stimuli) or across all velocities (for the three trapezoid stimuli). Both plots show the same effect: both the maximum PFR_EE_/PFR_Isol_ ratio and the average PFR_EE_/PFR_Isol_ ratio occurred in Layers 2 and Upper 3, with a systematic decrease with depth to L5 (Figures 8, 9). Both plots also indicate that while the EE-induced increase in response magnitude decreased with cortical depth, responses in EE animals always remain higher than those in animals housed in isolation.

#### Spontaneous firing rates in multi-neuronal clusters

**Figure 10:**
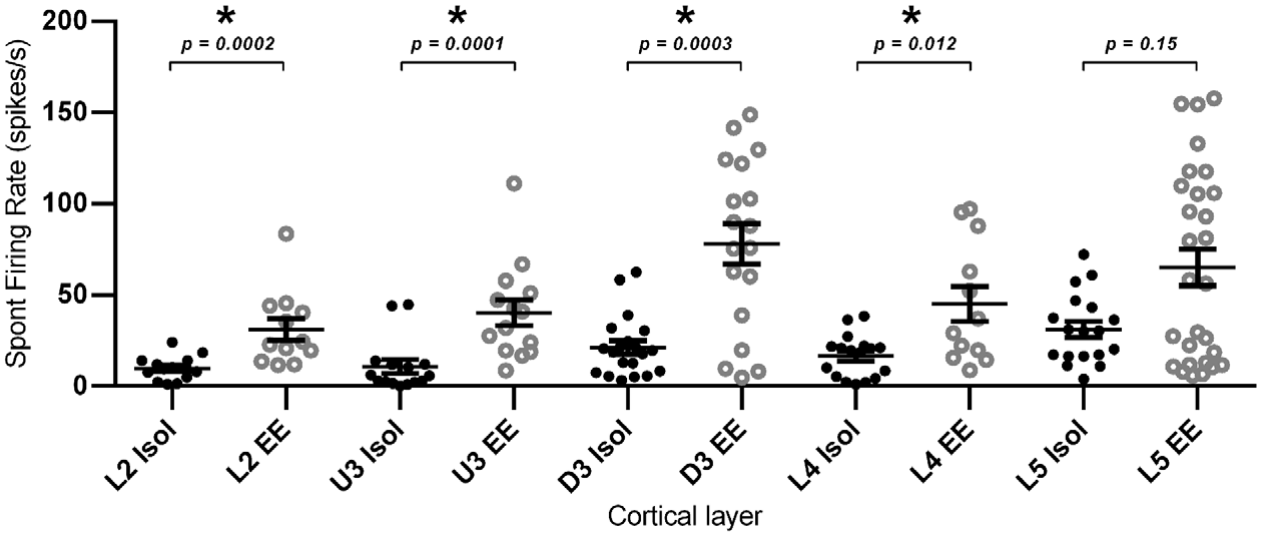
**Elevated spontaneous firing rate following EE**. Spontaneous firing rate was measured in the 200 ms window prior to stimulus onset, and was extracted from multi-neuronal clusters for EE (*n* = 84 clusters) and Isolated (*n* = 84 clusters) animals. The figure plots the mean spontaneous firing rate in clusters grouped according to cortical layer. Significant increases in spontaneous firing rate were observed after EE in layers L2–L4, with no significant differences in spontaneous firing rates between the two housing conditions in L5. Data represents averages from all responsive clusters (±SEM) at all tested ramp velocities, separated by cortical lamina. ^*^p < 0.05.

Finally, in addition to the stimulus-driven activity, we examined if EE conditions altered spontaneous activity. For this calculation, average spontaneous cluster firing rates were obtained from each cluster in the 200 ms period prior to onset of the basic trapezoidal stimulus, for all cortical layers in the two housing conditions (Figure 10). As with the changes in stimulus-driven activity reported above, average spontaneous firing rates in EE animals were significantly higher (Mann–Whitney U *t*-test, *p* < 0.05; see Figure 10 for exact *p*-values) than those in Isolation-housed animals in L2–4 (Isolated vs. EE (spikes/s): L2 = 9.78 ± 1.75 vs. 31.13 ± 5.92; U3 = 10.81 ± 3.70 vs. 40.33 ± 7.09; D3 = 21.29 ± 3.70 vs. 77.91 ± 11.08; L4 = 16.49 ± 2.82 vs. 45.15 ± 9.55). In L5, there were no differences in spontaneous firing rates between the two housing conditions (Isolated vs. EE: 32.77 ± 5.16 vs. 71.28 ± 10.48 spikes/s, *p* > 0.05). The highest average spontaneous firing rate after EE was observed in D3.

## Discussion

In response to exposure to EE, barrel cortex neurons demonstrated a very large increase in responsiveness to sensory input without any changes in response timing, throughout all cortical laminae of barrel cortex, in response to simple and varied complex, naturalistic stimuli, across a variety of response patterns, and in both short-term responses (up to 50 ms post-stimulus onset for short-duration stimuli) and longer-lasting responses (up to 200 ms post-stimulus onset for a longer duration stimulus). Thus, the plasticity underlying the effects of EE appears to be exercised across lamina, stimulus type, response pattern, and response duration. Additionally, EE also caused an increase in spontaneous activity in all laminae except in Layer 5. EE provides animals with cognitive, sensory and motor stimulation, through interactions with a complex environment, increased physical activity, and social interactions. The combination of these factors is thought to be responsible for promoting cortical plasticity in normal animals (as used in our study) and in models of disease and deprivation (Diamond et al., 1993; Van Praag et al., 2000; Nithianantharajah and Hannan, 2006; Rema et al., 2006; Baroncelli et al., 2010). In the following discussion we restrict ourselves to studies of adult plasticity and its mechanisms as developmental plasticity may include mechanisms not available in adulthood or much attenuated in adulthood (viz. Lendvai et al., 2000; Rajan and Irvine, 2010).

### Excitation/inhibition balance after EE and sensory cortical responsiveness

EE is generally reported to increase sensory cortical responsiveness in sensory cortex studies restricted to recordings from some layers. Mainardi et al. (2010) found that evoked potentials were increased in layers 3/4 of visual cortex of rats exposed to EE and, at the single neuron level, enrichment resulted in no change in RF size or spontaneous activity (Beaulieu and Cynader, 1990a) but increased responsiveness, orientation tuning, and temporal contrast tuning (Beaulieu and Cynader, 1990b, layers unknown). In primary auditory cortex (A1), 8 weeks of auditory enrichment resulted in increased stimulus-evoked neuronal response rates, spontaneous rate, and response latency, in layer IV (Engineer et al., 2004), increased auditory evoked potential amplitudes (Engineer et al., 2004; Percaccio et al., 2005, 2007) and decreased ability to follow high stimulus repetition rates (Percaccio et al., 2005, 2007). We too found increases in driven and spontaneous activity but no change in response latency. In other studies in adult auditory cortex, different types of enhanced auditory experience distorts the tonotopic map (Pienkowski and Eggermont, 2009; Zhou et al., 2011) and the ability to follow high stimulus rates (Zhou et al., 2011). However, there is dispute as to whether it decreases neuronal frequency selectivity at all frequencies (Zhou et al., 2011) or increases it within the frequency band of the enrichment stimulus (Pienkowski and Eggermont, 2009). In non-primary sensory cortex (posterior auditory field) EE increased response strength and decreased RF size, leading to an increase in spectral and temporal selectivity (Jakkamsetti et al., 2012). In contrast, Polley et al. (2004) reported that naturalistic sensory experience in their EE conditions decreased neuronal responses and RF size in barrel cortex, but only in the upper layers and not in layer 4. However, (Guic et al., 2008) suggested that the effects in the Polley et al. (2004) study reflected the effects of habituation even despite EE, and a lack of active exploration; Guic et al. (2008) found that EE caused an increase in RF size in contrast to Polley et al. (2004). Megevand et al. (2009) report increased greater whisker-driven evoked potentials in barrel cortex after EE.

A variety of mechanisms may contribute to cortical plasticity after EE (reviews by Van Praag et al., 2000; Nithianantharajah and Hannan, 2006), including structural and molecular changes like changes in dendritic structure and function and enhanced synaptic plasticity (Yang et al., 2009; Fu and Zuo, 2011; Jung and Herms, 2012), increases in certain neurotransmitters (Giovannini et al., 2001; Naka et al., 2002) or in synaptic release probability (Percaccio et al., 2005), and shifts in cortical E/I ratios (Coq and Xerri, 1998; Engineer et al., 2004; Polley et al., 2004; Baroncelli et al., 2011), given that a decrease in inhibition can trigger plasticity, even in non-critical periods (Maya Vetencourt et al., 2008; Southwell et al., 2010; Maya-Vetencourt et al., 2012), through changes in neuronal connectivity (Baroncelli et al., 2010, 2012). Our results can most parsimoniously be explained by this last factor (not excluding that the other mechanisms may also contribute to this factor)—that EE-induced plasticity in our case was due to a shift in the E/I balance to favor E. The stability of neuronal connections in the adult brain is held to be due to maturation of cortical inhibitory interneurons which results in decreased plasticity (Fagiolini and Hensch, 2000; Hensch and Fagiolini, 2005). Induction of adult cortical plasticity appears then to be brought about primarily through a decrease in cortical inhibitory activity (Hensch and Fagiolini, 2005; Sale et al., 2007; Benali et al., 2008; Baroncelli et al., 2010, 2011; Luz and Shamir, 2012). Consistent with this idea, EE causes a reduction in the number of visual cortical neurons expressing GAD67 (Scali et al., 2012; Tognini et al., 2012), and a decrease in extracellular basal GABA levels, coupled with a restoration of white matter long-term potentiation (WM-LTP), suggesting decreased cortical inhibition (Sale et al., 2007). Decreases in GABAergic inhibition after EE, through a reduction in expression of GABA_A_ receptor subunits, have been demonstrated in adult rat auditory cortex (Zhou et al., 2011), and in cat visual cortex enrichment decreases the number of inhibitory synapses (Beaulieu and Colonnier, 1987). Treatment with IGF-1, a peptide thought to play a role in EE-induced plasticity by enhancing synaptic plasticity (Torres-Aleman, 1999; Aberg et al., 2000), causes a decrease in basal inhibitory neurotransmitter levels with no overall changes in basal glutamate levels (Maya-Vetencourt et al., 2012) and a shift in ocular dominance as measured by visual evoked potentials, indicating visual cortical plasticity in adult rats that had undergone monocular deprivation (Maya-Vetencourt et al., 2012).

The hypothesis that EE simply decreases cortical inhibition is not universally accepted: Nichols et al. (2007) found that GABA_A_ receptor-mediated inhibitory postsynaptic currents do not change in supragranular auditory cortex after EE but there was an increase in AMPA-mediated current amplitudes. Further, the group had previously shown that EE results in a decreased ability of AI neurons to follow rapid stimuli (Percaccio et al., 2005), inconsistent with effects predicted by a decrease in inhibition. Similarly, Zhou et al. (2011) reported that enhanced auditory experience decreased expression of GABA_A_ *and* NMDA receptors, but had no effect on the AMPA glutamate receptor.

Our extracellular recordings do not allow direct measurements of inhibition but the large increase in driven and spontaneous response strength strongly supports the inference that EE in our study also caused a shift in the cortical E/I ratio to favor excitation over inhibition. Sensory deprivation also induces cortical plasticity through a decrease in inhibition (Welker et al., 1989; Buonomano and Merzenich, 1998). Kelly et al. (1999) reported that whisker trimming leads to a loss of inhibitory input from surrounding whiskers, as well as a net decrease in tonic cortical inhibition, resulting in increased barrel cortex responses to the PW. In the mature barrel cortex, deprivation-induced plasticity decreases L4 feed-forward excitation to L2/3 inhibitory neurons but with improved inhibition to L2/3 pyramidal cells (House et al., 2011), resulting in E/I balance being maintained in L2/3. We found increased activity in all layers, indicating that an E/I balance was *not* maintained in L2/3. Thus, EE, in our case, appears to exert global cross-lamina effects primarily, if not solely, directed to favoring excitatory over inhibitory inputs. This does not mean that large *changes* in excitation need have occurred. It has been elegantly demonstrated that during normal processing in somatosensory cortex, excitation and inhibition are highly synchronized and well-correlated in time and strength, in a continuous manner (Okun and Lampl, 2008); thus, even small changes in timing and strength of the two processes could result in a very large increase in excitation as we see (Isaacson and Scanziani, 2011).

Only certain forms of inhibition may be affected after exposure to EE since, even in our extracellular recordings, we do see some indications of inhibition after EE, with decreases in post-stimulus responses to levels below pre-stimulus spontaneous activity (see Figure 4A). Jakkamsetti et al. (2012) suggest that EE affects basal levels of GABA and evoked GABA differently, such that stimulus-evoked inhibition remains intact, while basal inhibitory activity is suppressed after EE, which could explain our evidence of intact post-stimulus inhibition but increased spontaneous activity.

Cortical inhibition is heterogeneous, either acting to affect neuronal responses (Wehr and Zador, 2003; Haider et al., 2013) by increasing response selectivity and hence, RF sizes, or altering response gain (Isaacson and Scanziani, 2011; Wilson et al., 2012), including acting through “silent” shunting inhibition (Isaacson and Scanziani, 2011). Selective loss of some forms of cortical inhibition may be all that is required to obtain the EE-induced increases in cortical spontaneous and driven responses. In auditory cortex, loss of surround inhibition can occur without loss of stimulus-driven within-field inhibition (Rajan, 1998, 2001)and then results in no change cortical in map topography (Rajan, 1998) but an increase in bandwidth of cortical neurons (Rajan, 2001), and thereby likely an increase in the overall representation of any specific frequency and increase response rates to the “best” frequency (Rajan, 1998). This parallels the observations that that post-stimulus inhibition is preserved in EE animals (our present study), and that EE does not change the exclusive representation area of a whisker in barrel cortex though there is an expansion of the total representation area of that whisker (Guic et al., 2008).

### Locus of EE-induced plasticity

Our finding of a global increase in neuronal responsiveness in barrel cortex after EE without response timing changes suggests an intra-cortical locus for plasticity (cf. Fox et al., 2002; Polley et al., 2004), especially given this pattern of effects even in the thalamo-recipient Layer 4 responses. Long-term whisker deprivation increases the functionality of specific GABA_A_ receptors on Layer 4 cells such that there is faster decay of inhibition in these cells (Li et al., 2009). In this case, a normal thalamic input could produce larger responses in Layer 4 as we find with EE. Our findings of an inverse relationship between cortical depth and the amount of EE induced increase in responses can be explained by postulating that a Layer 4 change is amplified by local mechanisms when information flows from Layer 4 to upper layers (Armstrong-James et al., 1992; Jellema et al., 2004; Lubke and Feldmeyer, 2007; Megevand et al., 2009), as has been suggested for experience-dependent plasticity in barrel cortex (Fox et al., 2002). This is consistent with the fact that supra-granular cortical layers show longer-lasting and greater amounts of plasticity than layers 4 or 5 (Glazewski and Fox, 1996; Wallace and Fox, 1999; Fox et al., 2002; Nichols et al., 2007). It is also consistent with the postulate (Nichols et al., 2007) that layer 2/3 is a “privileged substrate” for consolidating EE-induced cortical plasticity, possibly through spike-timing-dependent plasticity, or even structural plasticity, with studies reporting an EE-induced increase in dendritic branching in supragranular layers of the occipital cortex (Volkmar and Greenough, 1972; Greenough et al., 1973). A similar depth-dependent effect has been seen for plasticity evoked by rhythmic whisker stimulation in anesthetized animals, a paradigm suggested to engage the same intra-cortical plasticity mechanisms as EE (Megevand et al., 2009).

The absence of changes in response timing, as would be expected for plasticity evoked sub-cortically, is consistent with studies showing that thalamic plasticity is limited in the adult brain (Fox et al., 1996, 2002), except in the case of peripheral nerve injury (Li et al., 1995; Jones and Pons, 1998). However, it must be recognized that the debate on sub-cortical contributions to cortical changes in EE is not yet resolved. EE is capable of enhancing thalamocortical transmission in adult rat visual cortex (Mainardi et al., 2010), while EE in an experimental model of adult monocular amblyopia causes an increase in presynaptic thalamo-cortical activity, hence increasing postsynaptic stimulation of the visual cortex (Tognini et al., 2012). In the somatosensory cortex, whisker deprivation increases neuronal response rates in cortical Layer 4, with no changes in response rates in thalamic VPM (Wallace and Fox, 1999). In the auditory system it has been argued that cortical changes induced by enriched auditory experience are likely to occur in thalamus but be modified by local mechanisms in cortex (Pienkowski and Eggermont, 2009).

We are currently conducting further studies to investigate the exact mechanisms underlying the EE-induced changes in neuronal plasticity reported in the present study, using slice preparations and immunohistochemical techniques.

### Pattern of EE induced changes in responses to different behaviorally relevant stimuli

Our results show a stimulus amplitude-dependent increase in peak firing rates, where for all stimuli (except one) EE induced greater increases in excitatory responses at lower stimulus amplitudes than at higher stimulus amplitudes. The effect may simply reflect response saturation at higher levels; a multiplier effect will produce a larger change at lower stimulus amplitudes where responses are not saturated than it would at higher levels where responses are closer to saturation. This is consistent with the observation of Engineer et al. (2004) that stimuli that elicited smaller responses which did not reach saturation were more suitable to reveal whether EE has an effect on auditory cortical activity.

The odd one out of the stimuli in this inverse relationship between EE-induced increases and stimulus parameter (amplitude or velocity) was the stimulus waveform mimicking whisker motions seen in exploratory, free-whisking behavior. For this stimulus, the EE effect was non-monotonic with greatest effects at intermediate amplitudes than at higher amplitudes where EE effects either plateaued or even decreased in relative potency compared to the EE effects at intermediate amplitudes. EE influences exploratory behavior (Dell'omo et al., 2000; Frostig, 2006), thus making a stimulus that mimics exploratory whisker motion most relevant for investigation of EE effects. It is interesting to speculate that the maximization of EE effects to particular amplitudes of free, exploratory whisking may be linked to the effects of EE on exploratory behaviors—does EE promote rat behavior such that the animals are more likely to explore objects when the object is at a particular distance from the animal, i.e., does the animal tend to position itself so that it can best explore an object at a certain distance away? Szwed et al. (2006) have shown that spike rate is particularly important in encoding (in trigeminal ganglion neurons) radial distance of an object and whisker exploration of environmental objects and the EE-induced optimization of firing rate at particular amplitudes may be seen to support this hypothesis. However, against this is that an object a certain fixed distance from the animal will be at different radial distances along the length of whiskers arrayed across the rostro-caudal axis of the mystacial pad. Thus, the relevance of the EE-induced increase in firing rate at particular deflection amplitudes for this specific stimulus alone remains unknown.

### Novelty of objects and habituation

One final point that merits consideration is that it is generally held that novel objects in EE housing should be changed regularly to prevent habituation. We did not do so but still found highly elevated response rates, especially in supragranular layers, whereas Polley et al. (2004) reported that EE led to decreased responses in upper layers of barrel cortex (but not in layer 4), and a decrease in RF sizes. As noted above, Guic et al. (2008) suggested that the Polley et al. (2004) results reflected the effects of habituation and a lack of active exploration even despite EE conditions. If this criticism is valid, it would indicate that, in our case, the constancy of the objects in the EE housing was not a deterrent to active exploration of those objects, insofar as such active exploration led to the observed increase in response rates to all stimuli. In part this may reflect the fact that, in our study, when cages were cleaned and food and water replenished, the experimenters made a point of bringing back to the surface all objects that had become submerged under the bedding material. It is possible that this re-appearance of the objects contributed to maintaining active exploration of the objects and thereby contributed to the effects observed in this study. Under such conditions the effects of EE appear to be remarkably well-expressed in barrel cortex, across all layers, stimulus types, response patterns, and response durations.

### Conflict of interest statement

The authors declare that the research was conducted in the absence of any commercial or financial relationships that could be construed as a potential conflict of interest.

## Supplementary material

Table S1 **Results of Two-Way repeated measures ANOVA statistical analysis of firing rate (PFR) and Latency to Peak (L_PFR_) in clusters responsive to the trapezoidal stimulus from 5 to 50 ms from stimulus onset (related to Figures 1B,C).** The table lists *F* statistics and degrees of freedom for both significant and non-significant factors for main and interaction terms.

Table S2 **Results of Two-Way repeated measures ANOVA statistical analysis of firing rate (PFR) and Latency to Peak (L_PFR_) in clusters responsive to the onset response of the object contact stimulus from 5 to 50 ms from stimulus onset (related to Figures 2B,C).** The table lists *F* statistics and degrees of freedom for both significant and non-significant factors for main and interaction terms.

Table S3 **Results of Two-Way repeated measures ANOVA statistical analysis of firing rate (PFR) and Latency to Peak (L_PFR_) in clusters responsive to the smooth surface discrimination whisker motion stimulus from 5 to 30 ms from stimulus onset (related to Figures 3B,C).** The table lists *F* statistics and degrees of freedom for both significant and non-significant factors for main and interaction terms.

Table S4 **Results of Two-Way repeated measures ANOVA statistical analysis of firing rate (PFR) and Latency to Peak (L_PFR_) in clusters responsive to the rough surface discrimination whisker motion stimulus from 5 to 30 ms from stimulus onset (related to Figures 4B,C).** The table lists *F* statistics and degrees of freedom for both significant and non-significant factors for main and interaction terms.

Table S5 **Results of Two-Way repeated measures ANOVA statistical analysis of firing rate (PFR) in clusters responsive to the exploratory “free whisking” stimulus from 5 to 200 ms from stimulus onset (related to Figure 6A); and firing rate (PFR) and Latency to Peak (L_PFR_) from 5 to 50 ms from stimulus onset (related to Figures 6B,C).** The table lists *F* statistics and degrees of freedom for both significant and non-significant factors for main and interaction terms.
