# Supplementary material for: Environmental enrichment causes a global potentiation of neuronal responses across stimulus complexity and lamina of sensory cortex
Source: Front Cell Neurosci. 2013 Aug 8;7:124. doi: 10.3389/fncel.2013.00124 (PMC3737482; doi:10.3389/fncel.2013.00124)
Supplement: Table S1 — Results of Two-Way repeated measures ANOVA statistical analysis of firing rate (PFR) and Latency to Peak (LPFR) in clusters responsive to the trapezoidal stimulus from 5 to 50 ms from stimulus onset (related to Figures 1B,C). The table lists F statistics and degrees of freedom for both significant and non-significant factors for main and interaction terms. [file 56456__Data_Sheet_1.DOCX]

**Supplementary Data**

**Table S1. Results of Two-way repeated measures ANOVA statistical analysis of firing rate (PFR) and Latency to Peak (**L_PFR_**) in clusters responsive to the trapezoidal stimulus from 5-50ms from stimulus onset (related to Fig. 1B & C).** The table lists F statistics and degrees of freedom for both significant and non-significant factors for main and interaction terms.

| Response metric: Peak excitatory firing rate (PFR) in the onset response analysis window from 5-50 ms from stimulus onset**.** | | |
| --- | --- | --- |
| **Layer** | **Main terms** | **Interaction terms** |
| L2 | Group *F*_1,25_ = 8.04, *p* = 0.009  Velocity *F*_2,50_ = 9.66, *p* = 0.0003 | Velocity x Group *F*_2,50_ = 5.13, *p* = 0.009 |
| U3 | Group *F*_1,27_ = 4.32, *p* = 0.047  Velocity *F*_2,54_ = 31.55, *p* < 0.0001 | Velocity x Group *F*_2,54_ = 2.86, *p* = 0.066 |
| D3 | Group *F*_1,37_ = 23.39, *p* < 0.0001  Velocity *F*_2,74_ = 28.83, *p* < 0.001 | Velocity x Group *F*_2,74_ = 0.61, *p* = 0.55 |
| L4 | Group *F*_1,27_ = 22.87, *p* < 0.001  Velocity *F*_2,54_ = 35.63, *p* < 0.0001 | Velocity x Group *F*_2,54_ = 0.40, *p =* 0.67 |
| L5 | Group *F*_1,44_ = 7.65, *p* =0.008  Velocity *F*_2,88_ = 101.4, *p* < 0.0001 | Velocity x Group *F*_2,88_ = 6.14, *p* = 0.003 |
|  | | |
| Response metric: Latency to PFR in the onset response analysis window from 5-50 ms from stimulus onset**.** | | |
| **Layer** | **Main terms** | **Interaction terms** |
| L2 | Group *F*_1,25_ = 0.069, *p* = 0.79  Velocity *F*_2,50_ = 5.46, *p* = 0.007 | Velocity x Group *F*_2,50_ = 0.29, *p =* 0.75 |
| U3 | Group *F*_1,27_ = 0.003, *p* = 0.96  Velocity *F*_2,54_ = 9.13, *p* = 0.0004 | Velocity x Group *F*_2,54_ = 0.16, *p* = 0.85 |
| D3 | Group *F*_1,37_ = 2.82, *p* = 0.10  Velocity *F*_2,74_ = 12.76, *p* < 0.0001 | Velocity x Group *F*_2,74_ = 1.33, *p* = 0.27 |
| L4 | Group *F*_1,27_ = 0.54, *p* = 0.47  Velocity *F*_2,54_ = 15.06, *p* < 0.0001 | Velocity x Group *F*_2,54_ = 0.14, *p =* 0.87 |
| L5 | Group *F*_1,44_ = 0.84, *p* =0.34  Velocity *F* _2,88_ = 16.89, *p* < 0.0001 | Velocity x Group *F* _2,88_ = 2.27, *p* = 0.11 |
