# Supplementary material for: Environmental enrichment causes a global potentiation of neuronal responses across stimulus complexity and lamina of sensory cortex
Source: Front Cell Neurosci. 2013 Aug 8;7:124. doi: 10.3389/fncel.2013.00124 (PMC3737482; doi:10.3389/fncel.2013.00124)
Supplement: Table S2 — Results of Two-Way repeated measures ANOVA statistical analysis of firing rate (PFR) and Latency to Peak (LPFR) in clusters responsive to the onset response of the object contact stimulus from 5 to 50 ms from stimulus onset (related to Figures 2B,C). The table lists F statistics and degrees of freedom for both significant and non-significant factors for main and interaction terms. [file 56456__Data_Sheet_2.DOCX]

**Supplementary Data**

**Table S2. Results of Two-way repeated measures ANOVA statistical analysis of firing rate (PFR) and Latency to Peak (**L_PFR_**) in clusters responsive to the onset response of the object contact stimulus from 5-50ms from stimulus onset (related to Fig. 2B & C).** The table lists F statistics and degrees of freedom for both significant and non-significant factors for main and interaction terms.

| Response metric: Peak excitatory firing rate (PFR) in the onset response analysis window from 5-50 ms from stimulus onset**.** | | |
| --- | --- | --- |
| **Layer** | **Main terms** | **Interaction terms** |
| L2 | Group *F*_1,23_ = 10.47, *p* = 0.004  Amplitude *F*_9,207_ = 11.38, *p* < 0.0001 | Amplitude x Group *F*_9,207_ = 9.83, *p* < 0.0001 |
| U3 | Group *F*_1,30_ = 14.63, *p* = 0.0006  Amplitude *F*_9,270_ = 25.64, *p* < 0.0001 | Amplitude x Group *F*_9,270_ = 3.79, *p* = 0.0002 |
| D3 | Group *F*_1,33_ = 43.80, *p* < 0.0001  Amplitude *F*_9,297_ = 29.12, *p* < 0.001 | Amplitude x Group *F*_9,297_ = 2.89, *p* = 0.003 |
| L4 | Group *F*_1,27_ = 15.50, *p* = 0.0005  Amplitude *F*_9,253_ = 40.75, *p* < 0.0001 | Amplitude x Group *F*_9,253_ = 2.91, *p =* 0.003 |
| L5 | Group *F*_1,38_ = 3.92, *p* =0.055  Amplitude *F*_9,342_ = 48.61, *p* < 0.0001 | Amplitude x Group *F*_9,342_ = 3.58, *p* = 0.0003 |
|  | | |
| Response metric: Latency to PFR in the onset response analysis window from 5-50 ms from stimulus onset**.** | | |
| **Layer** | **Main terms** | **Interaction terms** |
| L2 | Group *F*_1,23_ = 1.46, *p* = 0.24  Amplitude *F*_9,207_ = 4.75, *p* < 0.0001 | Amplitude x Group *F*_9,207_ = 0.45, *p =* 0.91 |
| U3 | Group *F*_1,30_ = 0.010, *p* = 0.92  Amplitude *F*_9,270_ = 12.31, *p* < 0.0001 | Amplitude x Group *F*_9,270_ = 2.53, *p* = 0.0084 |
| D3 | Group *F*_1,33_ = 1.74, *p* = 0.20  Amplitude *F*_9,297_ = 14.05, *p* < 0.0001 | Amplitude x Group *F*_9,297_ = 1.10, *p* = 0.36 |
| L4 | Group *F*_1,27_ = 0.69, *p* = 0.41  Amplitude *F*_9,243_ = 48.12, *p* < 0.0001 | Amplitude x Group *F*_9,243_ = 3.62, *p =* 0.0003 |
| L5 | Group *F*_1,38_ = 6.71, *p* =0.014  Amplitude *F*_9,342_ = 37.17, *p* < 0.0001 | Amplitude x Group *F*_9,342_ = 0.55, *p* = 0.83 |
