# Supplementary material for: Environmental enrichment causes a global potentiation of neuronal responses across stimulus complexity and lamina of sensory cortex
Source: Front Cell Neurosci. 2013 Aug 8;7:124. doi: 10.3389/fncel.2013.00124 (PMC3737482; doi:10.3389/fncel.2013.00124)
Supplement: Table S3 — Results of Two-Way repeated measures ANOVA statistical analysis of firing rate (PFR) and Latency to Peak (LPFR) in clusters responsive to the smooth surface discrimination whisker motion stimulus from 5 to 30 ms from stimulus onset (related to Figures 3B,C). The table lists F statistics and degrees of freedom for both significant and non-significant factors for main and interaction terms. [file 56456__Data_Sheet_3.DOCX]

**Supplementary Data**

**Table S3. Results of Two-way repeated measures ANOVA statistical analysis of firing rate (PFR) and Latency to Peak (**L_PFR_**) in clusters responsive to the smooth surface discrimination whisker motion stimulus from 5-30ms from stimulus onset (related to Fig. 3B & C).** The table lists F statistics and degrees of freedom for both significant and non-significant factors for main and interaction terms.

| Response metric: Peak excitatory firing rate (PFR) in the onset response analysis window from 5-30 ms from stimulus onset**.** | | |
| --- | --- | --- |
| **Layer** | **Main terms** | **Interaction terms** |
| L2 | Group *F*_1,24_ = 13.07, *p* = 0.001  Amplitude *F*_9,216_ = 4.52, *p* < 0.0001 | Amplitude x Group *F*_9,216_ = 0.34, *p* = 0.96 |
| U3 | Group *F*_1,30_ = 14.47, *p* = 0.0007  Amplitude *F*_9,270_ = 49.39, *p* < 0.0001 | Amplitude x Group *F*_9,270_ = 0.38, *p* = 0.95 |
| D3 | Group *F*_1,31_ = 43.54, *p* < 0.0001  Amplitude *F*_9,279_ = 23.44, *p* < 0.001 | Amplitude x Group *F*_9,279_ = 3.34, *p* = 0.0007 |
| L4 | Group *F*_1,29_ = 26.52, *p* < 0.0001  Amplitude *F*_9,261_ = 71.65, *p* < 0.0001 | Amplitude x Group *F*_9,261_ = 9.00, *p* < 0.0001 |
| L5 | Group *F*_1,39_ = 4.74, *p* =0.036  Amplitude *F*_9,351_ = 60.00, *p* < 0.0001 | Amplitude x Group *F*_9,351_ = 2.81, *p* = 0.0034 |
|  | | |
| Response metric: Latency to PFR in the onset response analysis window from 5-30 ms from stimulus onset**.** | | |
| **Layer** | **Main terms** | **Interaction terms** |
| L2 | Group *F*_1,24_ = 2.43, *p* = 0.13  Amplitude *F*_9,216_ = 3.78, *p* = 0.0002 | Amplitude x Group *F*_9,216_ = 1.23, *p =* 0.28 |
| U3 | Group *F*_1,30_ = 0.047, *p* = 0.83  Amplitude *F*_9,270_ = 5.42, *p* < 0.0001 | Amplitude x Group *F*_9,270_ = 1.22, *p* = 0.29 |
| D3 | Group *F*_1,31_ = 0.056 *p* = 0.82  Amplitude *F*_9,279_ = 5.45, *p* < 0.0001 | Amplitude x Group *F*_9,279_ = 0.60, *p* = 0.80 |
| L4 | Group *F*_1,29_ = 0.22, *p* = 0.65  Amplitude *F*_9,261_ = 9.62, *p* < 0.0001 | Amplitude x Group *F*_9,261_ = 1.19, *p =* 0.30 |
| L5 | Group *F*_1,39_ = 0.26, *p* =0.61  Amplitude *F*_9,351_ = 29.45, *p* < 0.0001 | Amplitude x Group *F*_9,351_ = 1.73, *p* = 0.08 |
