# Supplementary material for: Environmental enrichment causes a global potentiation of neuronal responses across stimulus complexity and lamina of sensory cortex
Source: Front Cell Neurosci. 2013 Aug 8;7:124. doi: 10.3389/fncel.2013.00124 (PMC3737482; doi:10.3389/fncel.2013.00124)
Supplement: Table S4 — Results of Two-Way repeated measures ANOVA statistical analysis of firing rate (PFR) and Latency to Peak (LPFR) in clusters responsive to the rough surface discrimination whisker motion stimulus from 5 to 30 ms from stimulus onset (related to Figures 4B,C). The table lists F statistics and degrees of freedom for both significant and non-significant factors for main and interaction terms. [file 56456__Data_Sheet_4.DOCX]

**Supplementary Data**

**Table S4. Results of Two-way repeated measures ANOVA statistical analysis of firing rate (PFR) and Latency to Peak (**L_PFR_**) in clusters responsive to the rough surface discrimination whisker motion stimulus from 5-30ms from stimulus onset (related to Fig. 4B & C).** The table lists F statistics and degrees of freedom for both significant and non-significant factors for main and interaction terms.

| Response metric: Peak excitatory firing rate (PFR) in the onset response analysis window from 5-30 ms from stimulus onset**.** | | |
| --- | --- | --- |
| **Layer** | **Main terms** | **Interaction terms** |
| L2 | Group *F*_1,24_ = 13.26, *p* = 0.001  Amplitude *F*_9,216_ = 6.63, *p* < 0.0001 | Amplitude x Group *F*_9,216_ = 1.47, *p* = 0.16 |
| U3 | Group *F*_1,29_ = 29.78, *p* < 0.0001  Amplitude *F*_9,261_ = 26.13, *p* < 0.0001 | Amplitude x Group *F*_9,261_ = 0.23, *p* = 0.99 |
| D3 | Group *F*_1,31_ = 44.80, *p* < 0.0001  Amplitude *F*_9,279_ = 2.13, *p* < 0.001 | Amplitude x Group *F*_9,279_ = 0.37, *p* = 0.95 |
| L4 | Group *F*_1,27_ = 16.61, *p* = 0.0004  Amplitude *F*_9,243_ = 34.27, *p* < 0.0001 | Amplitude x Group *F*_9,243_ = 2.64, *p =* 0.006 |
| L5 | Group *F*_1,38_ = 3.92, *p* =0.055  Amplitude *F*_9,342_ = 48.61, *p* < 0.0001 | Amplitude x Group *F*_9,342_ = 3.58, *p* = 0.0003 |
|  | | |
| Response metric: Latency to PFR in the onset response analysis window from 5-30 ms from stimulus onset**.** | | |
| **Layer** | **Main terms** | **Interaction terms** |
| L2 | Group *F*_1,24_ = 0.29, *p* = 0.59  Amplitude *F*_9,216_ = 1.76, *p* = 0.077 | Amplitude x Group *F*_9,216_ = 0.83, *p =* 0.59 |
| U3 | Group *F*_1,29_ = 0.06, *p* = 0.81  Amplitude *F*_9,261_ = 1.38, *p* =0.20 | Amplitude x Group *F*_9,261_ = 5.18, *p* < 0.0001 |
| D3 | Group *F*_1,31_ = 0.14, *p* = 0.71  Amplitude *F*_9,279_ = 2.32, *p* = 0.016 | Amplitude x Group *F*_9,279_ = 1.60, *p* = 0.12 |
| L4 | Group *F*_1,27_ = 2.30, *p* = 0.14  Amplitude *F*_9,243_ = 10.45, *p* < 0.0001 | Amplitude x Group *F*_9,243_ = 0.31, *p =* 0.97 |
| L5 | Group *F*_1,38_ = 0.15, *p* =0.70  Amplitude *F*_9,342_ = 51.44, *p* < 0.0001 | Amplitude x Group *F*_9,342_ = 1.87, *p* = 0.06 |
