# Supplementary material for: Environmental enrichment causes a global potentiation of neuronal responses across stimulus complexity and lamina of sensory cortex
Source: Front Cell Neurosci. 2013 Aug 8;7:124. doi: 10.3389/fncel.2013.00124 (PMC3737482; doi:10.3389/fncel.2013.00124)
Supplement: Table S5 — Results of Two-Way repeated measures ANOVA statistical analysis of firing rate (PFR) in clusters responsive to the exploratory “free whisking” stimulus from 5 to 200 ms from stimulus onset (related to Figure 6A); and firing rate (PFR) and Latency to Peak (LPFR) from 5 to 50 ms from stimulus onset (related to Figures 6B,C). The table lists F statistics and degrees of freedom for both significant and non-significant factors for main and interaction terms. [file 56456__Data_Sheet_5.DOCX]

**Supplementary Data**

**Table S5. Results of Two-way repeated measures ANOVA statistical analysis of firing rate (PFR) in clusters responsive to the exploratory “free whisking” stimulus from 5-200ms from stimulus onset (related to Fig. 6A); and firing rate (PFR) and Latency to Peak (**L_PFR_**) from 5-50ms from stimulus onset (related to Fig. 6B & C).** The table lists F statistics and degrees of freedom for both significant and non-significant factors for main and interaction terms.

| Response metric: Peak excitatory firing rate (PFR) in the onset response analysis window from 5-200 ms from stimulus onset**.** | | |
| --- | --- | --- |
| **Layer** | **Main terms** | **Interaction terms** |
| L2 | Group *F*_1,24_ = 11.50, *p* = 0.0024  Amplitude *F*_9,216_ = 6.13, *p* < 0.0001 | Amplitude x Group *F*_9,216_ = 5.19, *p* < 0.0001 |
| U3 | Group *F*_1,27_ = 12.78, *p* = 0.0013  Amplitude *F*_9,243_ = 15.63, *p* < 0.0001 | Amplitude x Group *F*_9,243_ = 7.67, *p* < 0.0001 |
| D3 | Group *F*_1,31_ = 24.62, *p* < 0.0001  Amplitude *F*_9,279_ = 49.52, *p* < 0.0001 | Amplitude x Group *F*_9,279_ = 15.86, *p* < 0.0001 |
| L4 | Group *F*_1,27_ = 10.78, *p* = 0.0028  Amplitude *F*_9,243_ = 38.47, *p* < 0.0001 | Amplitude x Group *F*_9,243_ = 6.70, *p* < 0.0001 |
| L5 | Group *F*_1,37_ = 5.89, *p* = 0.0202  Amplitude *F*_9,333_ = 53.18, *p* < 0.0001 | Amplitude x Group *F*_9,333_ = 4.56, *p* < 0.0001 |
|  | | |
| Response metric: Peak excitatory firing rate (PFR) in the onset response analysis window from 5-50 ms from stimulus onset**.** | | |
| **Layer** | **Main terms** | **Interaction terms** |
| L2 | Group *F*_1,24_ = 18.07, *p* = 0.0003  Amplitude *F*_9,216_ = 9.99, *p* < 0.0001 | Amplitude x Group *F*_9,216_ = 7.19, *p* < 0.0001 |
| U3 | Group *F*_1,27_ = 7.30, *p* = 0.011  Amplitude *F*_9,243_ = 11.68, *p* < 0.0001 | Amplitude x Group *F*_9,243_ = 5.42, *p* < 0.0001 |
| D3 | Group *F*_1,31_ = 17.43, *p* = 0.0002  Amplitude *F*_9,279_ = 42.50, *p* < 0.0001 | Amplitude x Group *F*_9,279_ = 14.65, *p* < 0.0001 |
| L4 | Group *F*_1,27_ = 12.01, *p* = 0.0018  Amplitude *F*_9,243_ = 41.13, *p* < 0.0001 | Amplitude x Group *F*_9,243_ = 8.68, *p* < 0.0001 |
| L5 | Group *F*_1,37_ = 3.61, *p* =0.065  Amplitude *F*_9,333_ = 36.55, *p* < 0.0001 | Amplitude x Group *F*_9,333_ = 3.44, *p* = 0.0005 |
|  | | |
| Response metric: Latency to PFR in the onset response analysis window from 5-50 ms from stimulus onset**.** | | |
| **Layer** | **Main terms** | **Interaction terms** |
| L2 | Group *F*_1,24_ = 1.65, *p* = 0.21  Amplitude *F*_9,216_ = 1.49, *p* = 0.15 | Amplitude x Group *F*_9,216_ = 0.90, *p =* 0.53 |
| U3 | Group *F*_1,27_ = 0.45, *p* = 0.51  Amplitude *F*_9,243_ = 2.69, *p* = 0.0054 | Amplitude x Group *F*_9,243_ = 0.72, *p* = 0.69 |
| D3 | Group *F*_1,31_ = 3.16, *p* = 0.085  Amplitude *F*_9,279_ = 2.38, *p* = 0.013 | Amplitude x Group *F*_9,279_ = 1.94, *p* = 0.046 |
| L4 | Group *F*_1,27_ = 1.28, *p* = 0.27  Amplitude *F*_9,243_ = 2.76, *p* = 0.004 | Amplitude x Group *F*_9,243_ = 1.25, *p =* 0.27 |
| L5 | Group *F*_1,37_ = 0.70, *p* =0.41  Amplitude *F*_9,333_ = 3.61, *p* = 0.0003 | Amplitude x Group *F*_9,333_ = 0.80, *p* = 0.61 |
